# The evolution of antigen specific cancer immunotherapy from adoptive cell therapy to personalized cancer vaccines

**DOI:** 10.1007/s12672-026-05719-1

**Published:** 2026-08-05

**Authors:** Qingbo Chen, Sitong Yang, Haoyang Yu, Yingying He, Xiuping Guo, Ruohui Li, Lulu Wang

**Affiliations:** State Key Laboratory of Natural Medicines and Nuclear Medicine for Fundamental Research and New Drug Development, Beijing Key Laboratory of Key Technologies for Anti-Infective Drug Discovery and Applications, Institute of Medicinal Biotechnology, Chinese Academy of Medical Sciences, Beijing, 100050 China

**Keywords:** Immunotherapy, Tumor microenvironment, Chimeric antigen receptor, Immune checkpoint inhibitor, Vaccine

## Abstract

**Objective:**

This study systematically analyzed the literature on tumor antigen-based cancer immunotherapy by integrating quantitative bibliometric analysis with knowledge network visualization. It aimed to provide an overview of the developmental trajectory of this field, identify major research hotspots and key scientific challenges, and explore the underlying drivers of its evolution, thereby providing references for future antigen-targeted immunotherapy strategies and clinical translation.

**Methods:**

Literature related to tumor antigen-based cancer immunotherapy was retrieved from the Web of Science Core Collection, covering the period from September 15, 2005, to September 15, 2025. A total of 2,801 research articles were included. Bibliometric data were statistically analyzed using CiteSpace, VOSviewer, Charticulator, Scimago Graphica, and Bibliometrix, and visual scientific network maps were generated.

**Results:**

Bibliometric analysis showed that annual publication output in this field has remained relatively stable over the past six years, with approximately 170 publications per year and a peak of 188 publications in 2021. Regarding national contributions, the United States ranked first in publication output (1,282 publications), total citations (83,431 citations), and network centrality (0.46). Although China showed rapid growth in publication output and ranked second globally, its overall citation impact remained lower than that of several Western countries. In terms of academic influence, researchers such as Jeffrey Schlom from the National Cancer Institute and Chien-Fu Hung from Johns Hopkins University have maintained important positions through long-term research on tumor antigen recognition, immune regulation, and vaccine development. Temporal keyword analysis revealed an evolutionary transition from early adoptive immunotherapy-related studies, represented by terms such as “transfer therapy” and “adoptive immunotherapy”, to immune checkpoint regulatory strategies involving “nivolumab” and “immune checkpoint blockade”, and subsequently to emerging tumor antigen vaccine approaches characterized by “nanovaccine” and “mRNA vaccine”. Co-citation analysis further supported this evolutionary trajectory, indicating a gradual shift from broadly enhancing immune activation toward precise tumor antigen identification and optimization of antigen-driven immune responses.

**Conclusion:**

Over the past two decades, tumor antigen-based cancer immunotherapy has evolved from adoptive immune cell therapy and immune checkpoint regulation to personalized neoantigen vaccine strategies, forming a multi-level therapeutic framework involving antigen recognition, immune activation, and targeted delivery. Future advances in antigen prediction, sequencing technologies, mRNA vaccine platforms, and immune regulatory strategies may further promote the development of more precise, effective, and personalized cancer immunotherapies.

**Supplementary Information:**

The online version contains supplementary material available at https://doi.org/10.1007/s12672-026-05719-1.

## Introduction

Cancer is a group of diseases caused by the malignant proliferation of cells. Malignant cells not only consume host nutrients but also invade surrounding tissues and, in some cases, distant organs, eventually leading to systemic functional failure. Each year, cancer causes millions of deaths worldwide and remains a major threat to human health [1, 2]. Because cancer cells closely resemble normal cells and are prone to genetic mutation, achieving highly precise and selective tumor cell killing remains difficult, which substantially limits therapeutic efficacy. Although current cancer treatment has evolved into a multidisciplinary system that includes surgery, chemotherapy, radiotherapy, immunotherapy, and other therapeutic modalities, transforming some cancers from fatal diseases into manageable or treatable conditions and improving overall survival, existing treatments are still frequently associated with considerable adverse effects and cannot guarantee complete cure for all patients [3, 4]. Therefore, more definitive, targeted, and less toxic therapeutic strategies are still urgently needed to ultimately overcome cancer.

The immune surveillance system is an important line of defense for maintaining tissue homeostasis and recognizing abnormal cells. By identifying aberrantly expressed antigens on tumor cells, the immune system can activate antigen-specific immune responses and mediate tumor cell clearance. Accordingly, tumor antigen-based cancer immunotherapy, which is built around tumor antigen recognition and the regulation of immune effector responses, has gradually become an important direction in cancer treatment research. At present, antigen recognition-based therapeutic strategies have developed along several major paths, including adoptive cell therapy, immune checkpoint inhibitors, and tumor antigen vaccines, all of which have shown substantial value in clinical research and application [5–7].

Tumor antigens refer to molecules that are aberrantly expressed or overexpressed on tumor cells and serve as important markers for distinguishing malignant cells from normal cells. Among them, tumor-specific antigens and neoantigens are regarded as important targets for inducing precise antitumor immune responses because they can partially evade the constraints imposed by immune tolerance. In recent years, with advances in high-throughput sequencing, the understanding of immune recognition mechanisms, and antigen prediction technologies, research has gradually shifted from simply enhancing immune activation to precisely regulating the process of tumor antigen recognition. This shift has promoted the development of tumor antigen-based cancer immunotherapy toward a more individualized and precise direction [8–10].

However, the development of tumor antigen-based cancer immunotherapy still faces many challenges. The antigenic variability of tumor cells and the immunosuppressive microenvironment in which they reside severely restrict the targeting precision and therapeutic efficacy of existing treatments. Therefore, how to help immune cells penetrate this “fog” and more efficiently recognize and eliminate tumor cells remains a central challenge that urgently needs to be addressed in this field [11, 12].

Based on this background, the present study focuses on the development of tumor antigen-based cancer immunotherapy. Using bibliometric methods, we analyzed relevant publications from the Web of Science Core Collection (WoSCC) over the past two decades, with the aim of outlining the developmental trajectory of this field and identifying key turning points and their underlying drivers. By summarizing previous research trajectories, this study seeks to clarify future research patterns and academic directions in the field, thereby providing a reference for subsequent studies and supporting the continued development and advancement of tumor antigen-based cancer immunotherapy.

## Materials and methods

### Data collection and processing

The Web of Science Core Collection (WoSCC) was used as the retrieval database in this study to systematically analyze the developmental trends in tumor antigen-based cancer immunotherapy. Considering that tumor antigen recognition, antigen-specific immune activation, and antigen-targeted therapy constitute important foundations for the development of this field, antigen-related terms were incorporated into the cancer immunotherapy search strategy to focus on studies involving tumor antigen recognition and antigen-driven immune responses. The search strategy was as follows: TS = (cancer* OR tumor* OR neoplasm*) AND TS = (“antigenic specificity” OR “antigen specificity” OR “antigen specific” OR “antigen-specific” OR “ag-specific”) AND TS = (immunized OR immunity OR immune OR immunization) AND TS = (treat* OR therapy OR management).The search was conducted on September 15, 2025, and 2,801 relevant publications covering the period from 2005 to 2025 were included.

**Fig. 1:**
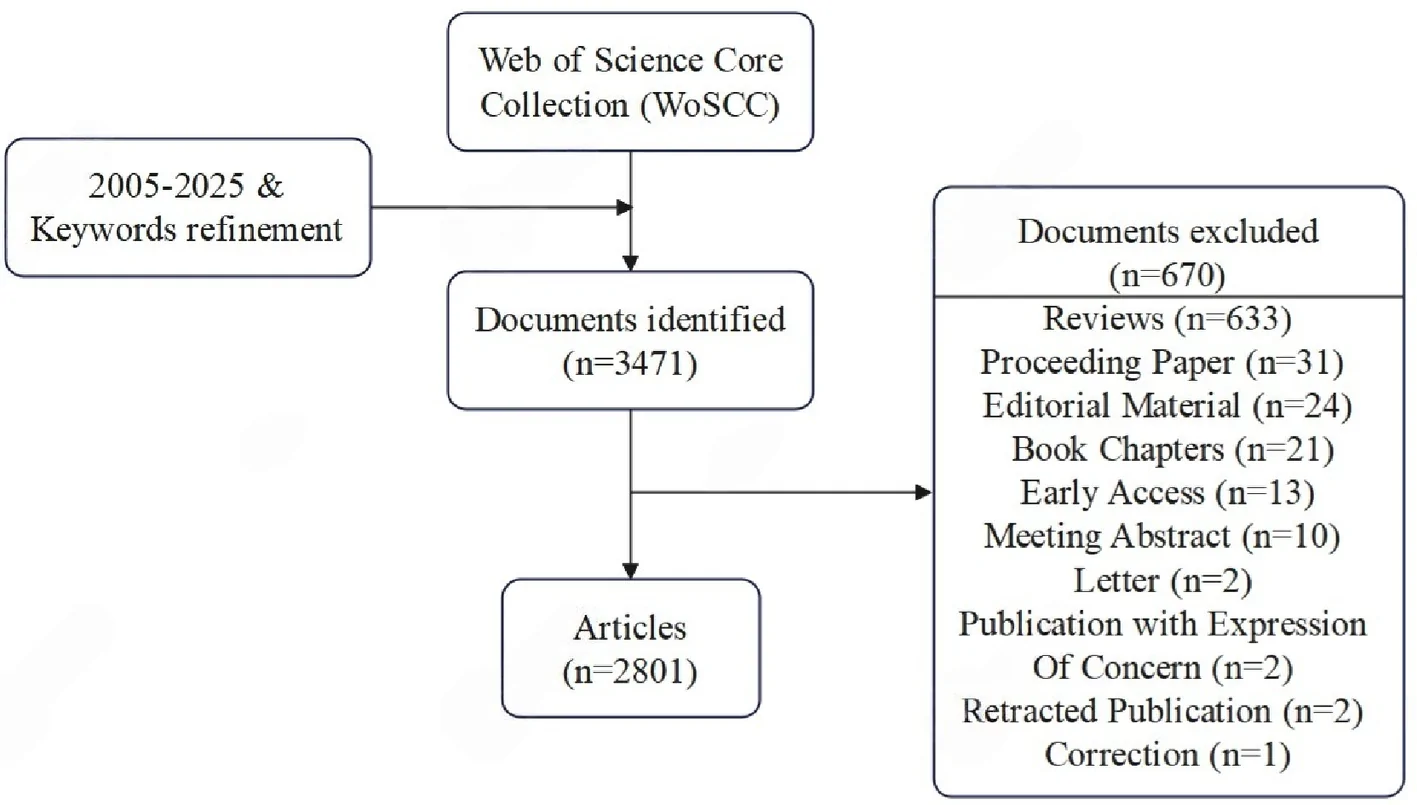
Flowchart of literature acquisition and screening

Only English-language research articles were included in this study. A total of 670 publications of other document types were excluded, including reviews (*n* = 633), proceeding papers (*n* = 31), editorial materials (*n* = 24), book chapters (*n* = 21), early access articles (*n* = 13), meeting abstracts (*n* = 10), letters (*n* = 2), publications with expressions of concern (*n* = 2), retracted publications (*n* = 2), and corrections (*n* = 1). The data collection and processing workflow is shown in Fig. 1.

### Bibliometric analysis and visualization

Bibliometric data processing and visualization were performed using VOSviewer 1.6.20, CiteSpace 6.4.R1, Microsoft Excel 2021, Scimago Graphica, Charticulator, and the Bibliometrix R package. Microsoft Excel was used to summarize the annual number of publications and related bibliometric data from 2005 to 2025, and to visualize changes in annual publication output and the overall growth trend of publications. VOSviewer was used to generate collaboration maps among countries and citation networks of journals. Scimago Graphica was applied to present national publication output and international collaboration patterns, while Charticulator was used to construct chord diagrams showing country-level publication output and collaboration. The Bibliometrix R package was used to generate author publication timeline plots and keyword trend maps.

In addition, CiteSpace, developed by Chaomei Chen and colleagues, was used to systematically analyze collaboration networks among authors, institutions, and journals, as well as keyword networks and document co-citation relationships. Corresponding visual knowledge maps were then generated. Through the integrated use of these bibliometric tools, this study provides a relatively comprehensive overview of the research landscape, knowledge structure, and developmental evolution of tumor antigen-based cancer immunotherapy, while also highlighting key nodes within the field [13, 14].

## Results

### Overview of research progress

**Fig. 2:**
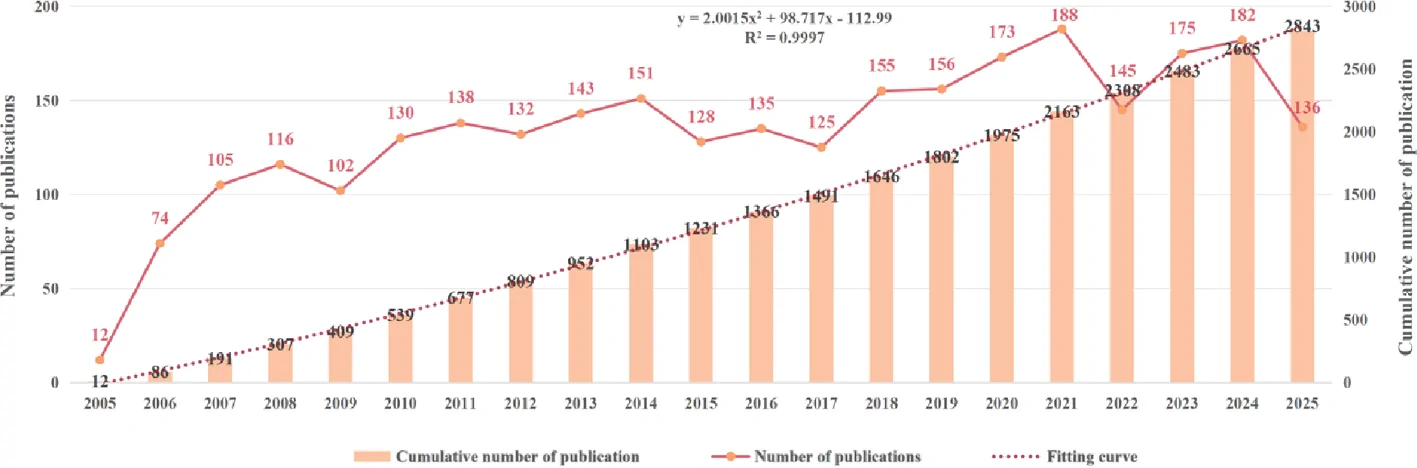
Annual publication volume

As shown in Fig. 2, the annual publication output in the field of tumor antigen-based cancer immunotherapy has undergone two major stages since 2005: a period of rapid growth followed by a phase of relatively stable output. Beginning with 12 publications in 2005, the field first experienced a five-year period of rapid expansion, with the annual number of publications reaching 130 by 2010. This was followed by a seven-year period of stable development, during which the annual output generally remained at a level comparable to that observed in 2010. After this period of stability, annual publication output began to increase again in 2018, marking the entry of the field into a new stage. Since then, the number of publications has gradually stabilized at approximately 170 articles per year and has remained at this level in recent years. It should be noted that the search in this study was conducted on September 15, 2025; therefore, the number of publications in 2025 represents only the output up to that date rather than complete annual data. To reflect the overall developmental trend up to 2025, the cumulative number of publications was estimated based on previous growth patterns and should be interpreted only as a trend reference.

**Fig. 3:**
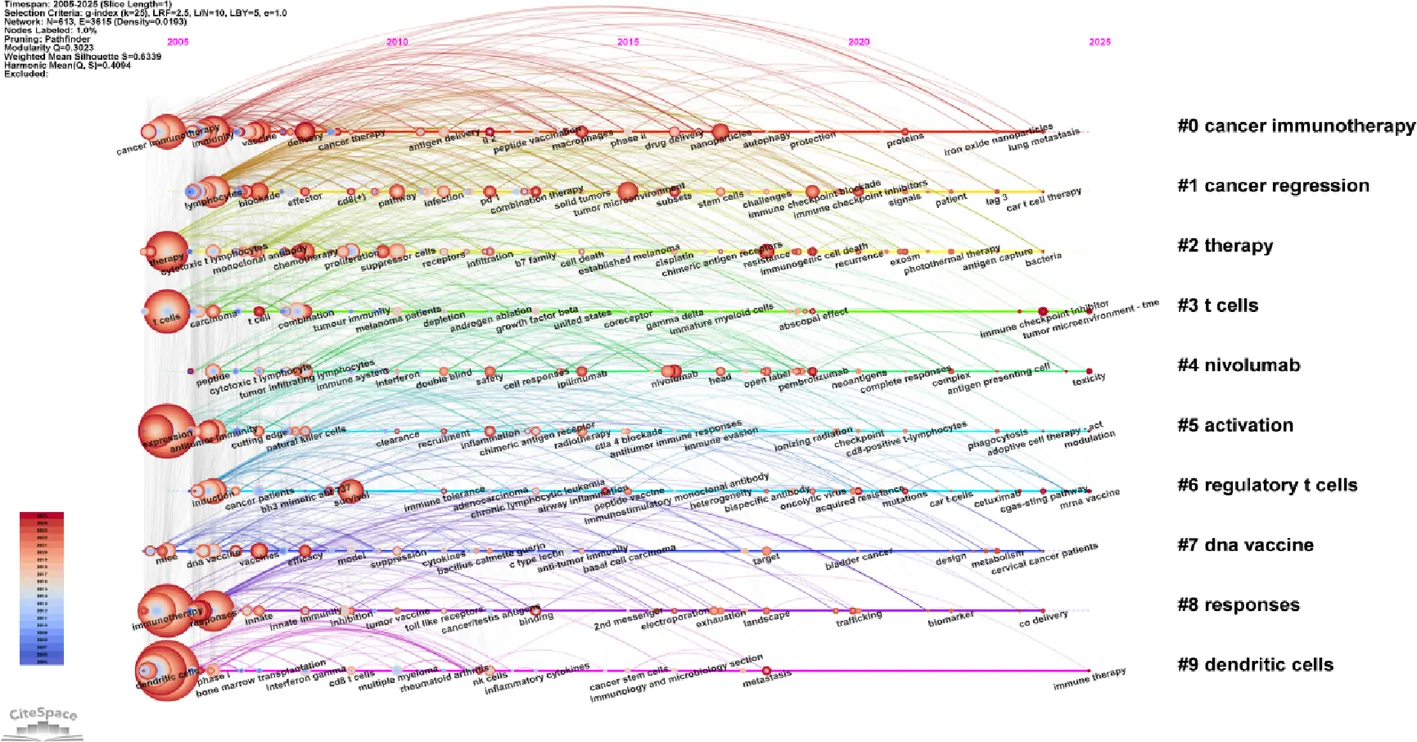
Timeline of keyword evolution

**Fig. 4:**
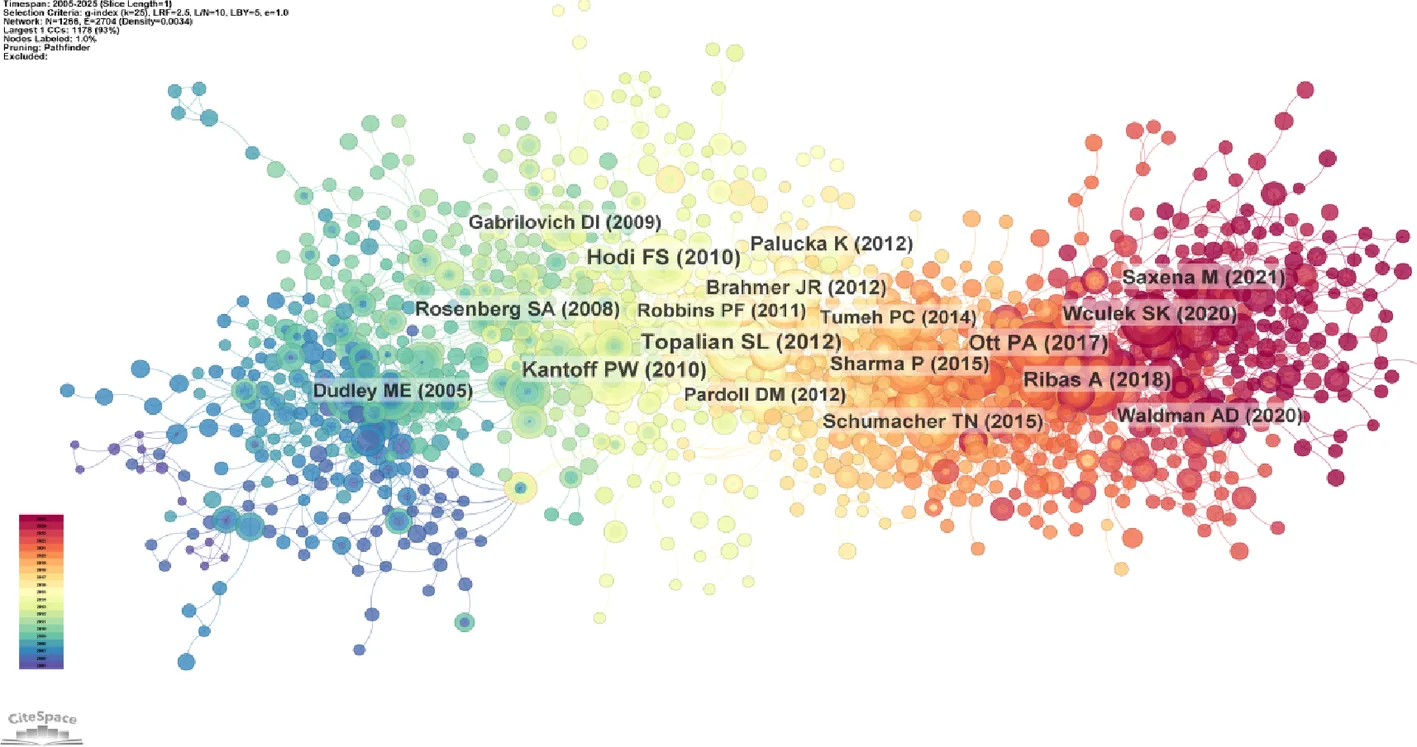
Literature co-citation network

Combined with the keyword timeline map (Fig. 3) and the document co-citation map (Fig. 4), the period from 2005 to 2010 can be regarded as a conceptual foundation stage for cancer immunotherapy. This stage was characterized by the gradual establishment of the theories of cancer immunosurveillance and immunoediting, as well as the emergence of the concept of T cell exhaustion in the context of chronic immune responses. At the same time, the mechanisms underlying immune checkpoint pathways, including CTLA-4 and PD-1, were progressively and systematically clarified. During this period, publication output in the field increased rapidly, rising by nearly one order of magnitude within five years.

In 2010, the publication of the phase III clinical trial results for the CTLA-4 antibody ipilimumab indicated not only that knowledge accumulation in the field had become increasingly mature, but also that the research focus had undergone a profound shift. Specifically, the field gradually moved from relying mainly on exogenous immune cells to exert antitumor effects toward restoring the host’s ability to recognize and eliminate tumor antigens by regulating immunosuppressive pathways [15]. Over the following seven years, studies on immune checkpoint inhibitors and their combination therapies developed rapidly. Although research activity remained high during this period, the dominant innovation paradigm was relatively stable, and annual publication output fluctuated around 130 articles.

In 2017, the first CAR-T cell therapy, Kymriah, was approved by the FDA, marking the formal entry of engineered adoptive immunotherapy based on artificial receptor-mediated antigen recognition into clinical application [16, 17]. This breakthrough further demonstrated the feasibility of achieving targeted tumor clearance by precisely regulating the antigen recognition capacity of immune cells, thereby promoting the development of tumor antigen-based cancer immunotherapy.

Meanwhile, the decreasing technical barriers to single-cell sequencing and other omics technologies substantially reduced the difficulty of studying tumor antigens. As a result, personalized neoantigen vaccines have gradually become a major hotspot in cancer immunotherapy and an important driving force behind the renewed increase in publication output in this field [18–20]. Driven by these two forces, research on tumor antigen-based cancer immunotherapy continued to grow steadily after 2018, with annual publication output remaining at approximately 170 articles in recent years.

### Country analysis

**Fig. 5:**
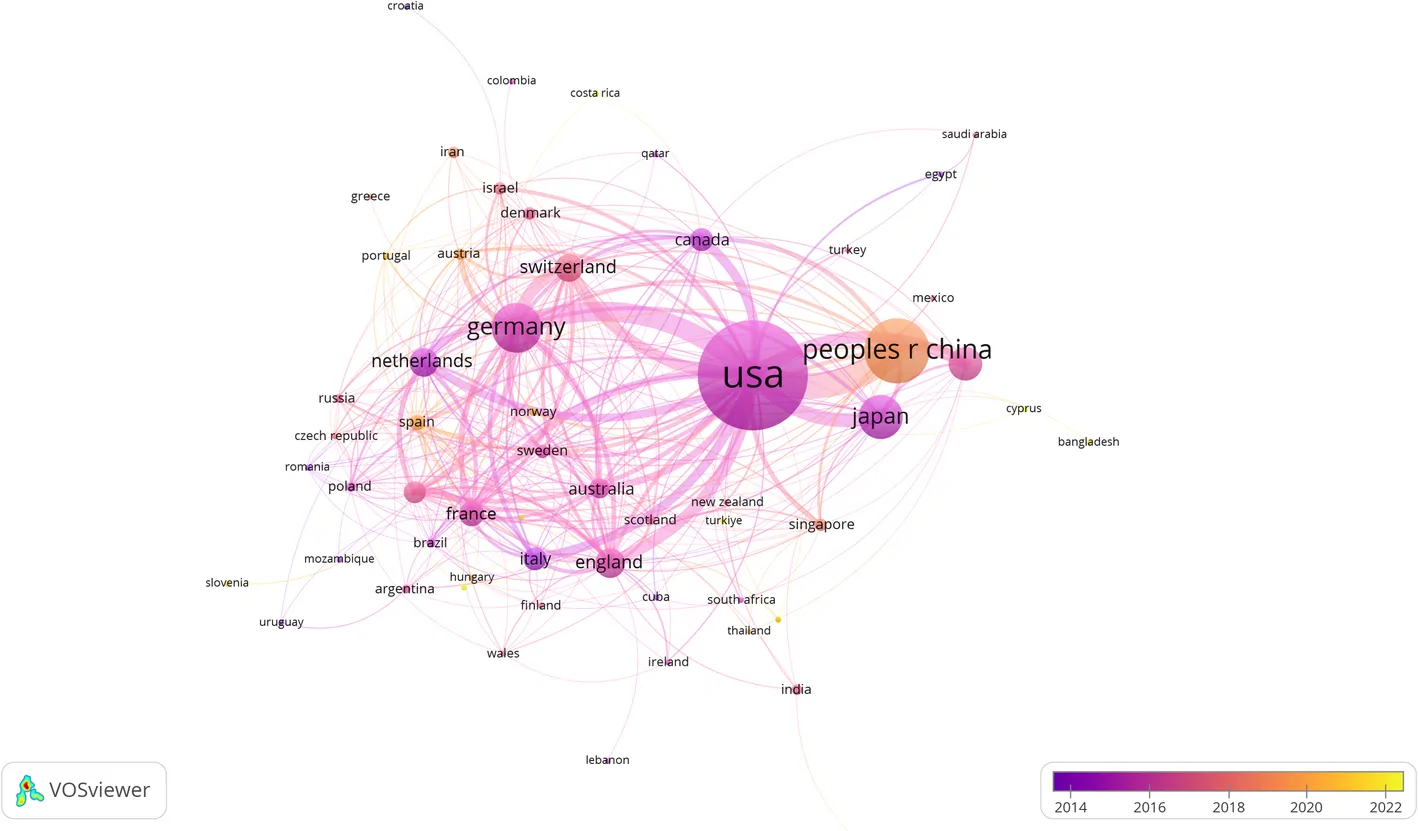
Time-zone view of international collaboration networks

A total of 62 countries published research articles related to tumor antigen-based cancer immunotherapy. As shown in Table 1, the United States occupied an important academic hub position in this field, with 1,282 publications, 83,431 citations, and a network centrality of 0.46. These findings indicate a high level of research communication activity and participation in international collaboration. China has shown rapid growth in publication output in recent years and contributed a substantial proportion of newly published studies during this period, as shown in Fig. 5. With 519 publications, China ranked second globally. This growth trend may partly reflect the continued increase in biomedical research investment and the rapid expansion of the research workforce in China in recent years.

Despite this marked increase in publication output, the average citation count of Chinese publications was 30.53, which was still considerably lower than that of the other countries among the top ten by publication volume. This phenomenon may be influenced by multiple factors, such as the publication of some studies in regional or emerging journals with relatively low citation rates, relatively limited participation in international collaboration networks, and differences in language and dissemination visibility, all of which may have affected the international citation performance of these publications to some extent [21, 22]. 

**Fig. 6:**
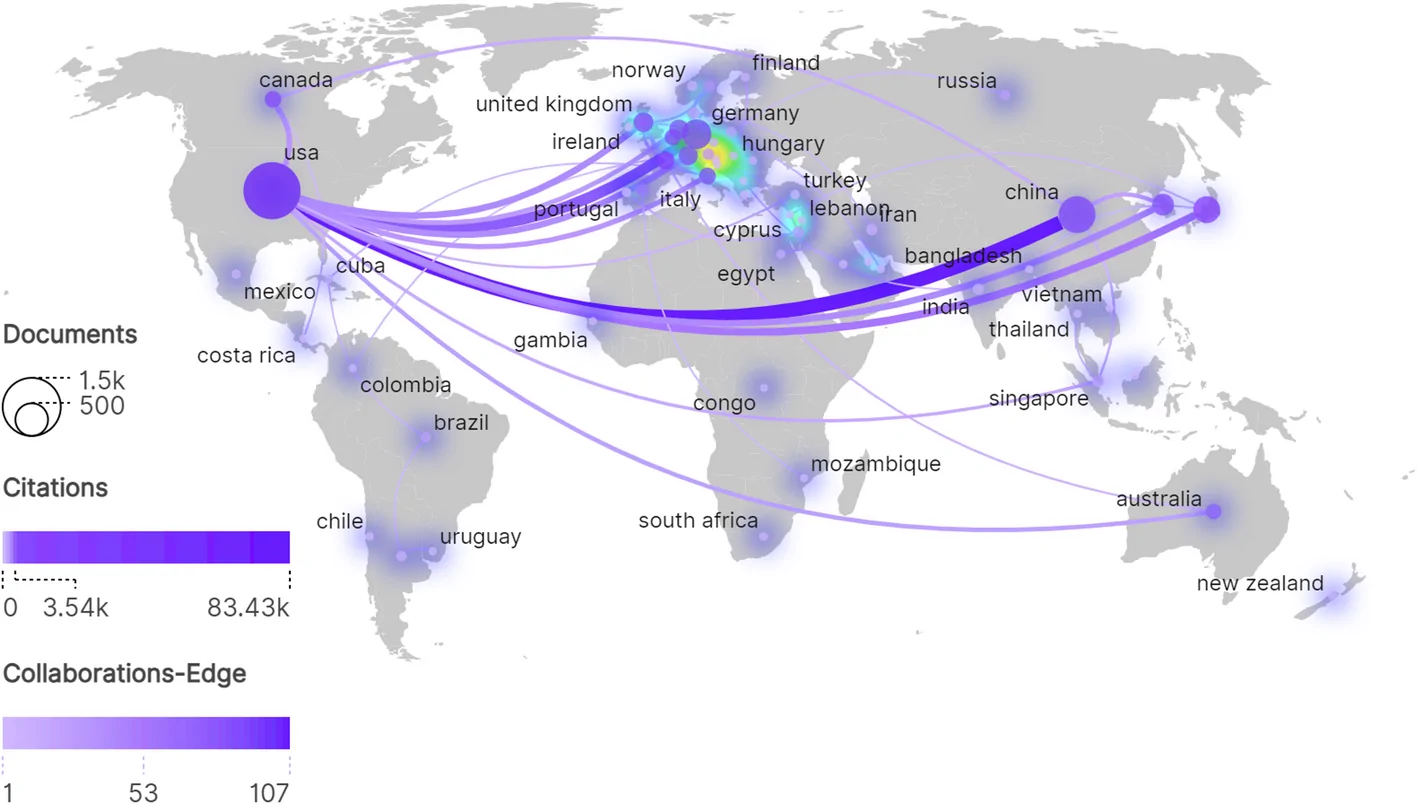
Geographical co-occurrence map of international collaboration networks

As shown in Table 1, European countries, including Germany, the United Kingdom, Netherlands, Switzerland, France, and Italy, also maintained important positions in terms of publication output in this field. Notably, the average citation counts of publications from these countries were generally high. This may be related to their earlier entry into the field, strong accumulation of basic research, and relatively mature clinical translation systems. The hotspot density distribution in Europe shown in Fig. 6 also partly supports the long-term and stable research activity of these countries in this field.

**Fig. 7:**
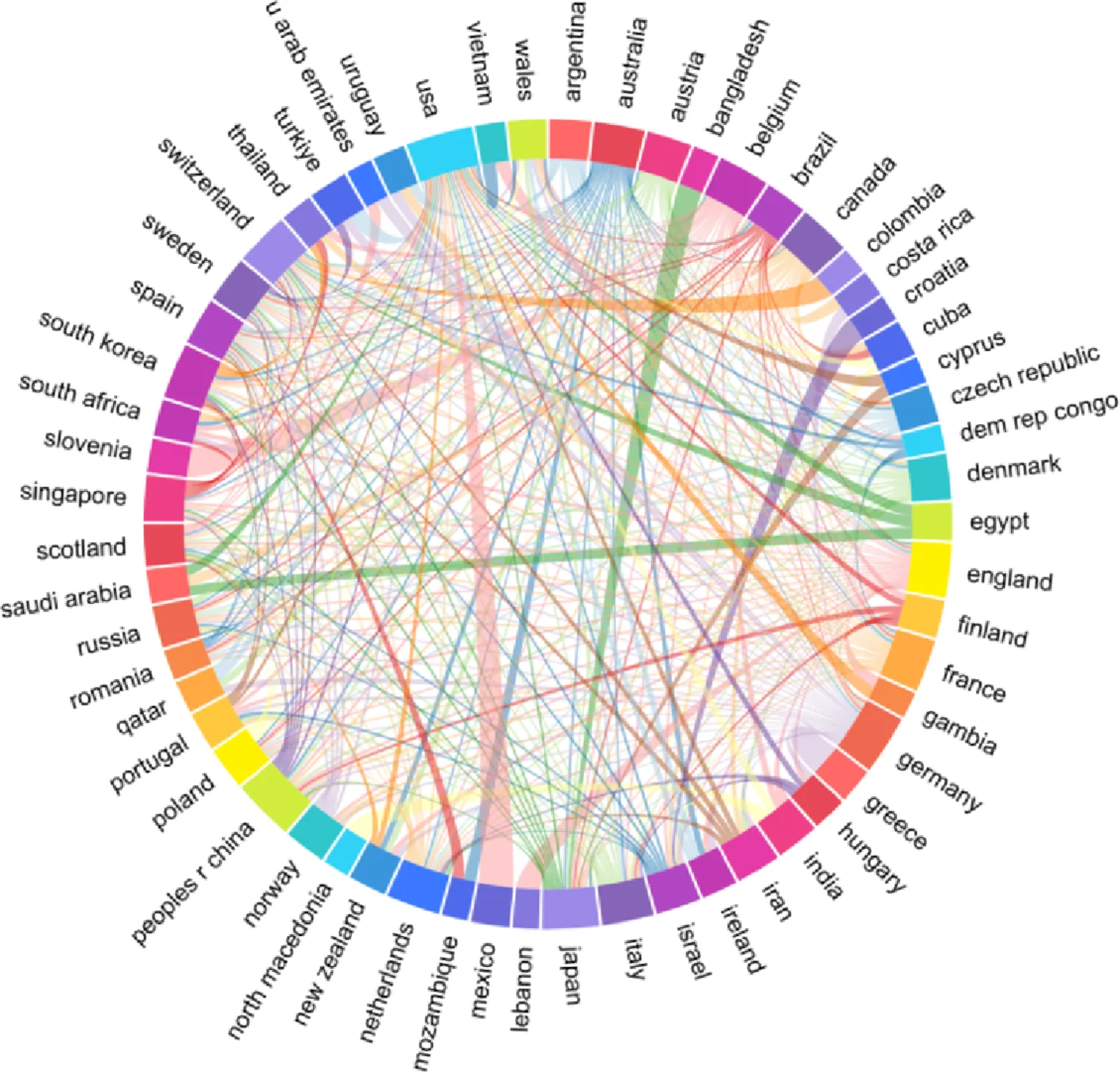
Chord diagram of international collaborations

In terms of the international collaboration network, the United States occupied a central position within the global research collaboration network and maintained collaborative links with multiple countries, indicating its high connectivity and important role in international research cooperation. As shown in Fig. 7, China, Japan, Germany, Iran, and other countries also actively participated in international collaboration and showed high levels of activity in regional and cross-regional cooperation. Together, these countries contributed to the formation of a multi-level and multicenter international research collaboration landscape.

**Table 1:** Top 10 countries by publication volume

Rank	Country	Publications	Citation count	Average citation count	Centrality
1	USA	1282	83,431	65.08	0.46
2	The People’s Republic of China	519	15,847	30.53	0.01
3	Germany	328	20,806	63.43	0.29
4	Japan	263	9435	35.87	0.1
5	South Korea	163	5427	33.29	0.01
6	England	131	6415	48.97	0.19
7	Netherlands	127	7870	61.97	0.02
8	Switzerland	119	6867	57.71	0.11
9	France	100	11,975	119.75	0.06
10	Italy	87	7015	80.63	0.03

### Author analysis

**Fig. 8:**
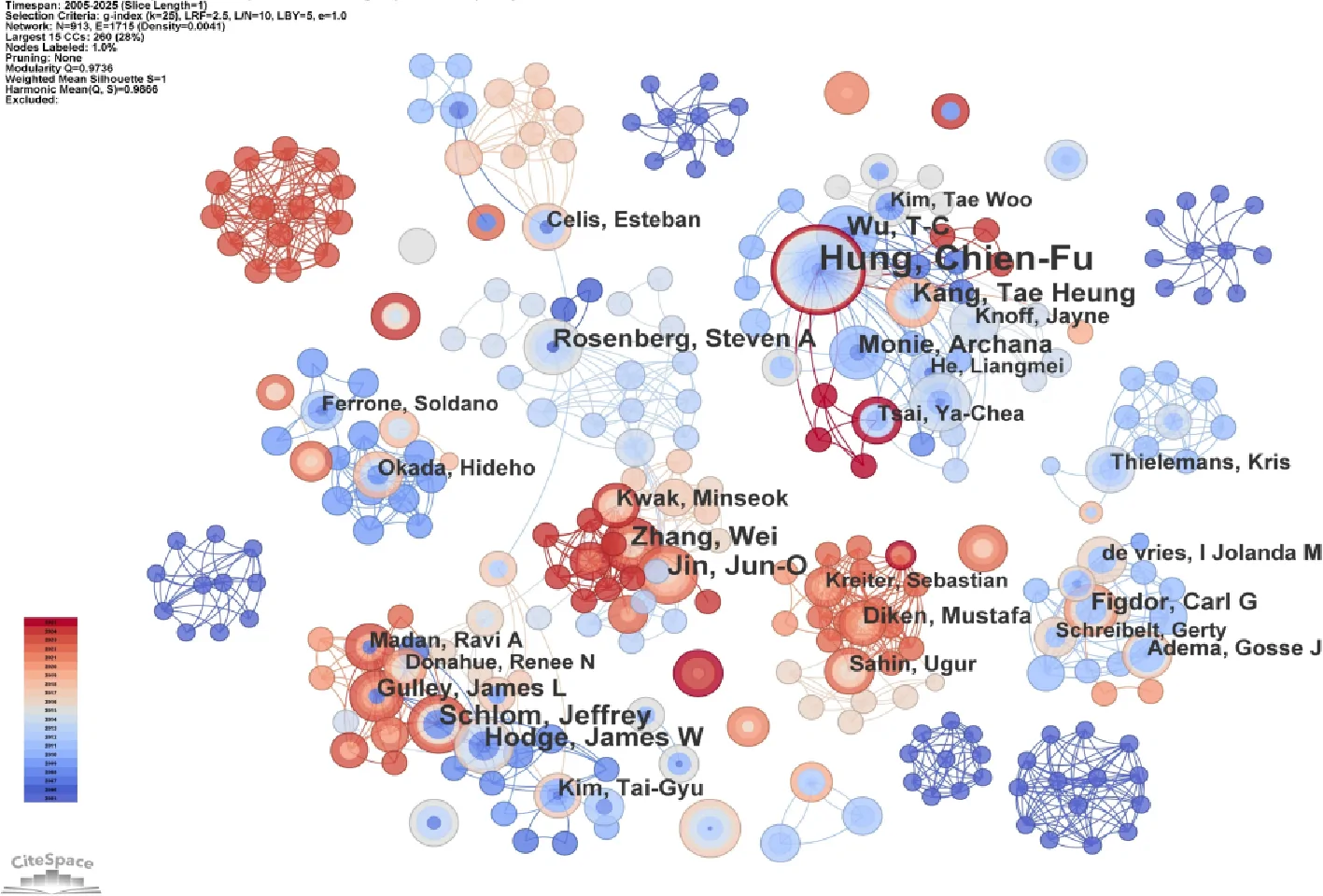
Author collaboration network

A total of 21,263 researchers have contributed to the field of tumor antigen-based cancer immunotherapy. Professor Chien-Fu Hung ranked first in publication output and third in citation count, with 38 articles and 1,184 citations. His research has mainly focused on HPV vaccines, vaccine adjuvants, immunotherapy, and related mechanistic investigations [23–26]. As shown in Table 2; Fig. 8, Professor Hung has made substantial academic contributions and has also played an important role in training researchers in this field. Members of his research team, including T.-C. Wu and Tae Heung Kang, ranked fourth and ninth in publication output, respectively, indicating their considerable academic influence in this area.

Professor Jeffrey Schlom and his team member James W. Hodge ranked second and eighth in publication output, respectively. Although their total number of publications was not the highest in the field, their articles showed relatively high average citation counts, reflecting broad recognition of their work on anticancer agents, cancer vaccines, and vaccine-related immune mechanisms [27–29]. Professor Douglas G. McNeel, who ranked third in publication output, had a smaller team size and fewer publications than the top two contributors; nevertheless, his research has made important contributions to the development of therapeutic strategies for prostate cancer [30, 31].

**Fig. 9:**
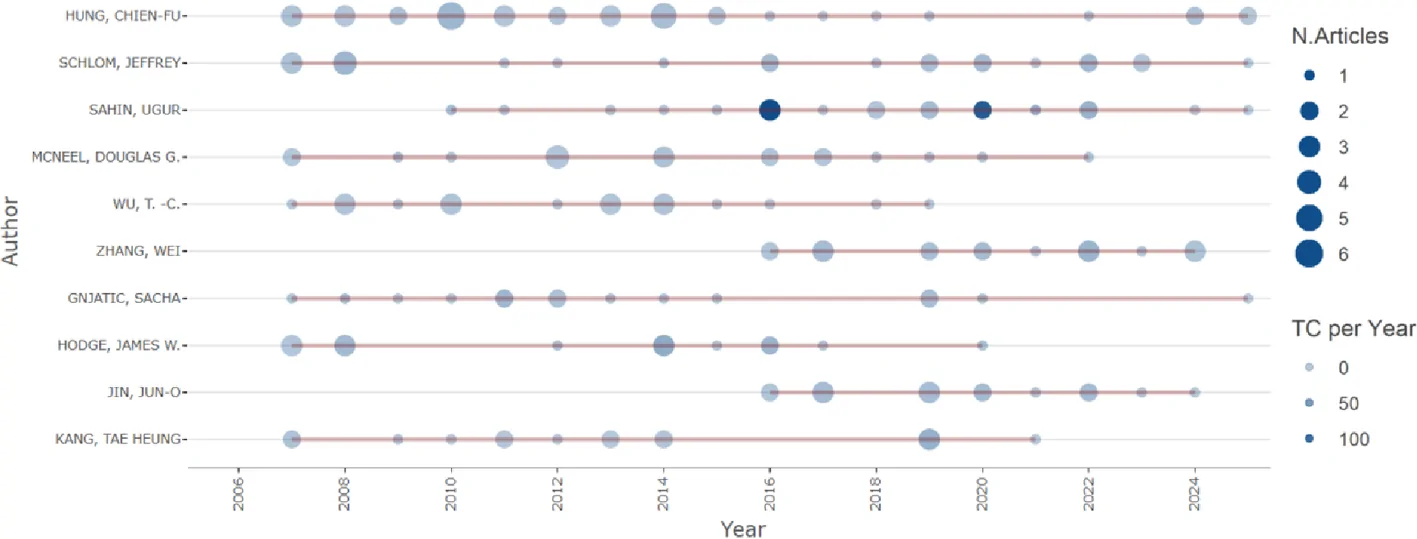
Timeline of author publication output

As shown in Fig. 9, Chinese scholars Wei Zhang and his collaborator Jun-O Jin have become increasingly active contributors in tumor antigen-based cancer immunotherapy in recent years. Their new attempts in tumor vaccine materials and delivery carriers have provided fresh perspectives for the development of this field [32–34]. More notably, although Professor Ugur Sahin did not rank among the most prolific authors in terms of publication output, his recent high-quality articles published in journals such as *Nature* and *Cancer Discovery* have received a large number of citations within a short period. With an H-index of 91, he has become one of the leading researchers in the field of RNA vaccine-based cancer therapy [35–37].

**Table 2:** Top 10 most productive author

Rank	Author	Publications	Citations	H-index
1	Hung, Chien-Fu	38	1184	54
2	Schlom, Jeffrey	23	1107	65
3	McNeel, Douglas G.	19	716	45
4	Wu, T.-C.	19	582	63
5	Zhang, Wei	17	507	24
6	Sahin, Ugur	16	668	91
7	Gnjatic, Sacha	15	1368	79
8	Hodge, James W.	15	1226	62
9	Kang, Tae Heung	15	577	33
10	Jin, Jun-O	15	480	39

### Institutional analysis

A total of 3,207 institutions contributed to publications in the field of tumor antigen-based cancer immunotherapy. As shown in Table 3, the National Cancer Institute at the National Institutes of Health (NCI), where Professor Jeffrey Schlom is affiliated, ranked first in both publication output and citation count, with 101 publications and 6,127 citations. As a national cancer research institution in the United States, the NCI has made substantial contributions to cancer prevention, diagnosis, treatment, and mechanistic research. Its high level of productivity may partly reflect the advantages of national research institutions in resource integration and multidisciplinary collaboration.

Johns Hopkins University, which is home to one of the world’s leading medical schools, ranked second among institutions with 61 publications. Approximately 62.3% of its publications in this field were contributed by Professor Chien-Fu Hung and his collaborators, suggesting that the research output of this institution in this area was, to some extent, driven by a core research team. In other words, a small number of highly productive research groups made a substantial contribution to the institution’s overall output. This phenomenon is relatively common in bibliometric analyses and reflects the clustering effect of core research groups within scientific output and academic collaboration networks.

**Fig. 10:**
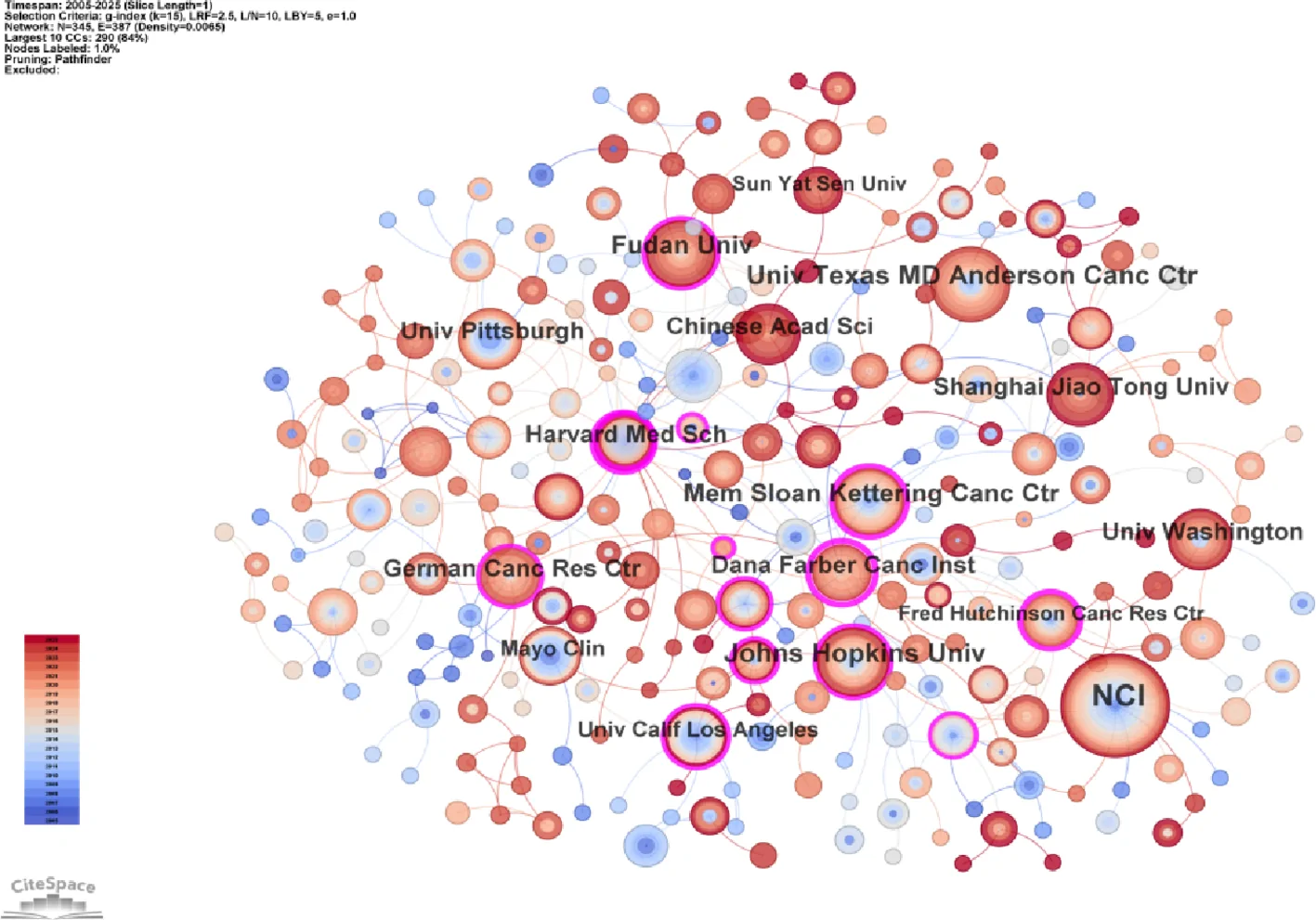
Institutional collaboration network

The University of Texas MD Anderson Cancer Center also showed high publication output and citation impact in this field, ranking third in institutional publication output. As shown in Fig. 10, Memorial Sloan Kettering Cancer Center, which ranked fifth in publication output, was not among the most prolific institutions but received 5,415 citations, indicating a high level of academic influence. In addition, this institution showed relatively high network centrality in the collaboration network, as indicated by the purple ring around the node, suggesting a relatively high level of connectivity and a central position within the international research collaboration network. This finding reflects its role as a potential collaboration hub. Similarly, Harvard Medical School and its affiliated hospital, Dana-Farber Cancer Institute, also played indispensable roles in researcher collaboration within this field.

In addition to institutions from the United States, several institutions from China and Germany were also observed to play important roles in this research area. Chinese institutions, including Fudan University, Shanghai Jiao Tong University, and the Chinese Academy of Sciences, appeared among the top ten institutions by publication output, reflecting their substantial research productivity in this field. The German Cancer Research Center also demonstrated an active presence in the international collaboration network, indicating its involvement in global research cooperation within cancer immunotherapy.

**Table 3:** Top 10 most productive institutions

Rank	Organization	Publications	Citations	H-index
1	National Cancer Institute	101	6127	51
2	Johns Hopkins University	61	5473	37
3	The University of Texas MD Anderson Cancer Center	61	4805	44
4	University of Washington	51	2568	25
5	Memorial Sloan Kettering Cancer Center	48	5415	32
6	University of Pittsburgh	47	3954	30
7	Fudan University	46	1257	22
8	Shanghai Jiao Tong University	45	1005	19
9	University of Pennsylvania	43	3703	26
10	Chinese Academy of Sciences	40	1187	22

### Journal analysis

The 2,801 articles included in this study were published in 558 journals, indicating a broad range of publication sources. As shown in Table 4, *Cancer Immunology*,* Immunotherapy* published 118 articles, accounting for 4% of the total number of articles, and ranked first in publication output in this field. It was followed by *Journal for Immunotherapy of Cancer*, with 113 articles, and *Clinical Cancer Research*, which ranked third with 94 articles. Overall, 70% of the top ten journals by publication output were specialized journals related to tumor immunotherapy. This finding suggests that the field has a strong disciplinary focus and has gradually formed a relatively stable publication pattern centered on specialized journals.

**Fig. 11:**
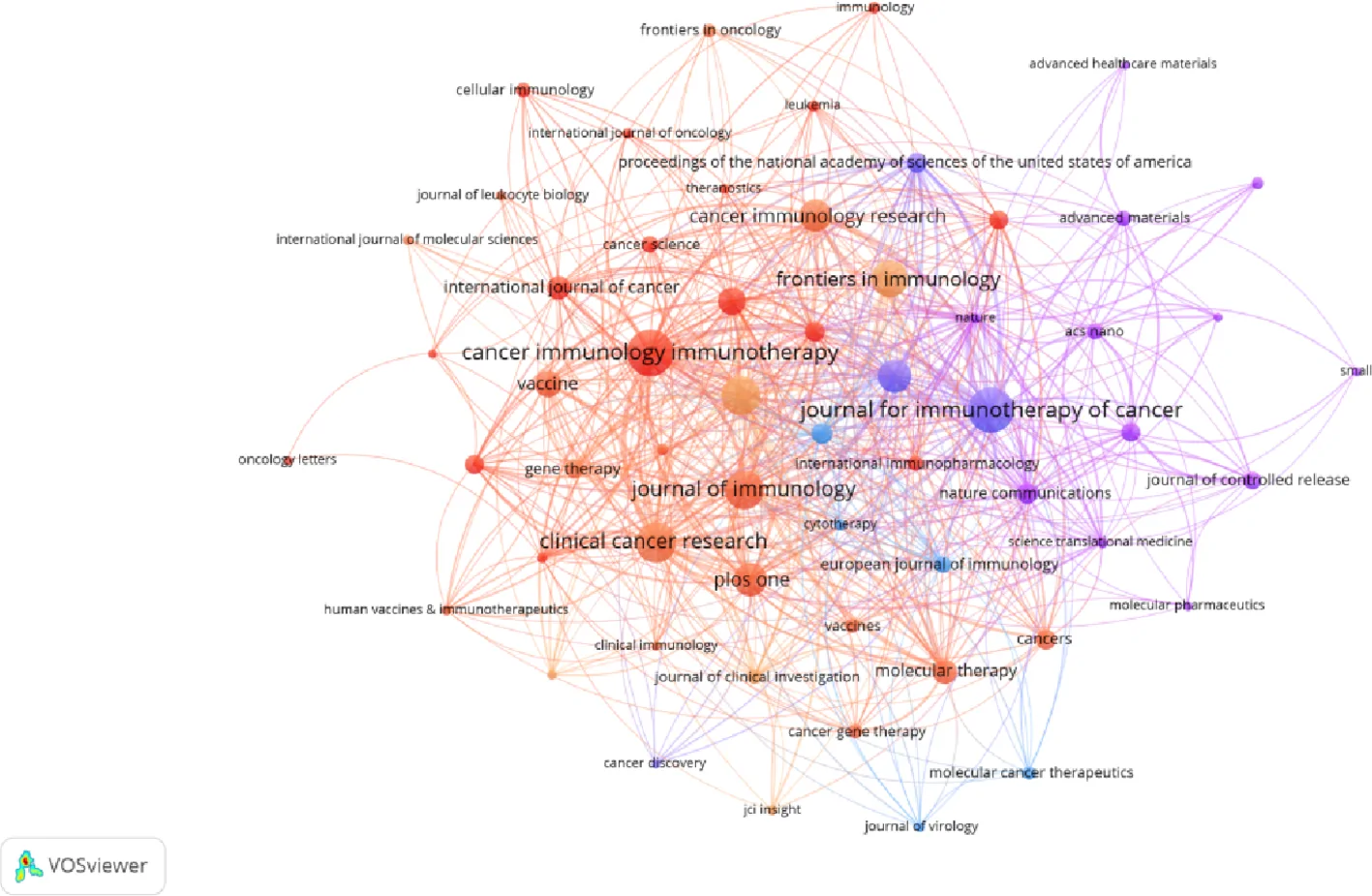
Network map of journal publication relationships

In the journal co-occurrence network shown in Fig. 11, relatively close citation relationships and collaborative connections can be observed among several tumor immunology-related journals, suggesting that a relatively stable academic communication system has been established in the field of tumor antigen-based cancer immunotherapy. Among the cited journals, more comprehensive journals tended to have higher citation counts. Although *Nature* did not publish a large number of articles in this field, its cumulative citation count reached 11,660, ranking among the top journals in this field. This indicates its strong capacity for interdisciplinary influence diffusion and suggests that *Nature* plays an important nodal role in disseminating key breakthrough studies.

**Fig. 12:**
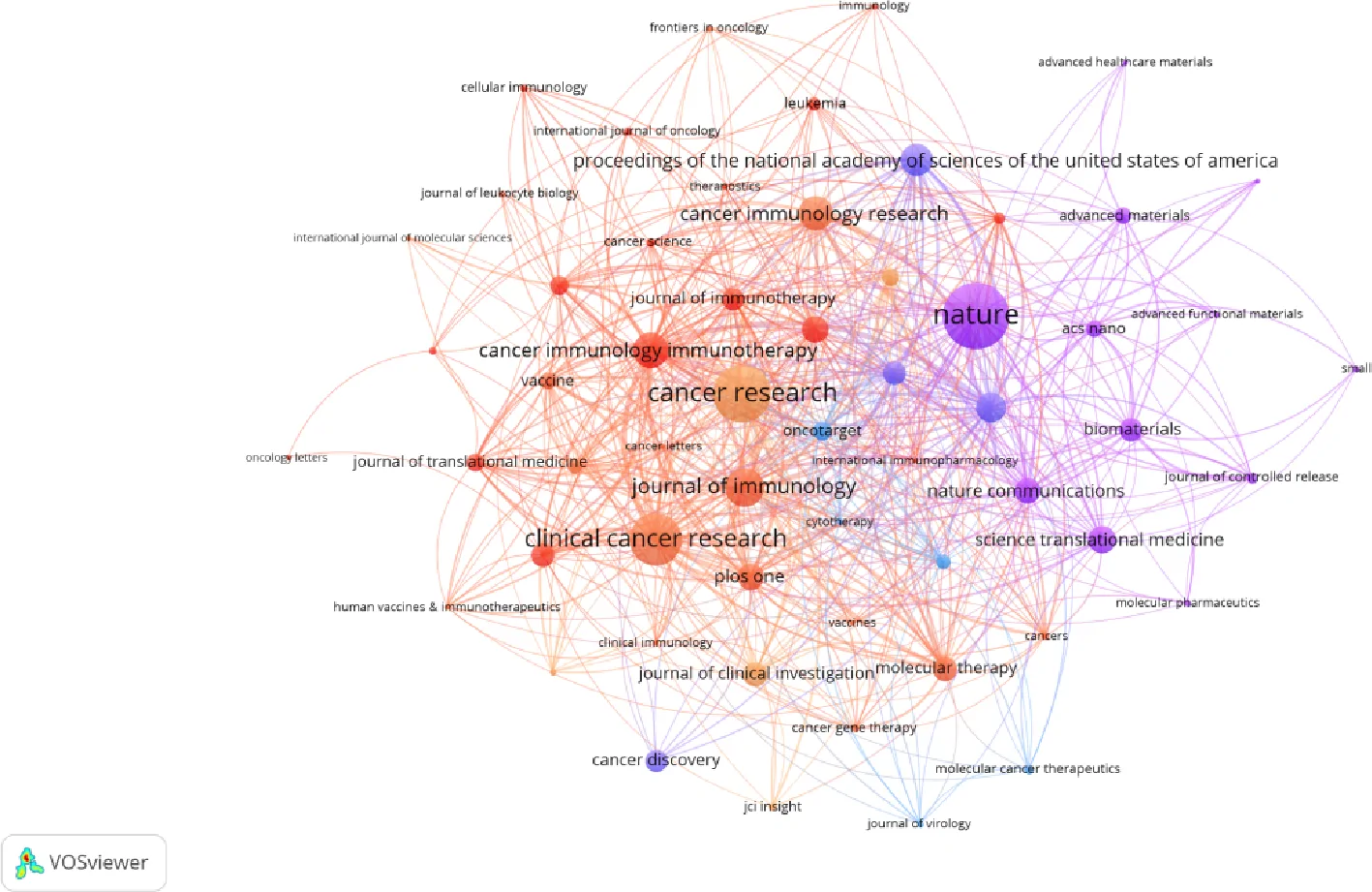
Network map of journal citation relationships

In addition, Fig. 12 clearly shows the high citation frequency of *Cancer Research* in this field, with 9,194 citations. While maintaining a relatively high publication output, this journal also demonstrated strong citation performance, suggesting that it has played an important role in knowledge integration related to tumor antigen research, immune mechanism elucidation, and therapeutic strategy exploration.

**Fig. 13:**
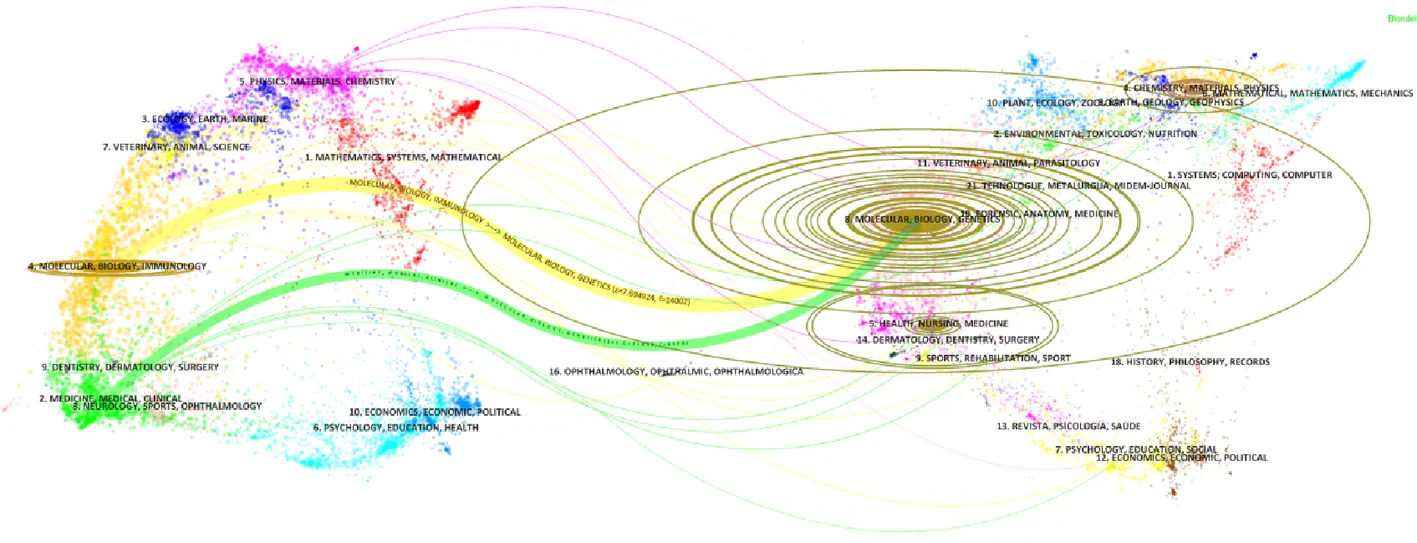
Dual-map overlay of journals

Figure 13 shows the citation and co-citation relationships among journal clusters. The clusters on the right represent cited journals, whereas those on the left represent citing journals. The connecting lines between the right and left clusters indicate the pathways of knowledge flow, and the thickness of each line represents the relative dominance of that pathway. A clear pattern can be observed in which knowledge flows from the “Molecular, Biology, Genetics” cluster on the right toward the “Molecular, Biology, Immunology” and “Medicine, Medical, Clinical” clusters on the left. This pattern reflects the gradual translation of basic research findings, such as antigen discovery and molecular mechanism elucidation, into immune regulation and clinical therapeutic strategies.

Although a relatively clear immunological and clinical medical knowledge system has been formed in this field, knowledge input from other disciplines, including “Physics, Materials, Chemistry”, “Molecular, Biology, Genetics”, and “Medicine, Medical, Clinical”, can still be observed. These findings indicate that tumor antigen-based cancer immunotherapy remains in a process of continuous integration and evolution, with its research boundaries being further expanded through multidisciplinary intersections [6, 38, 39].

**Table 4:**
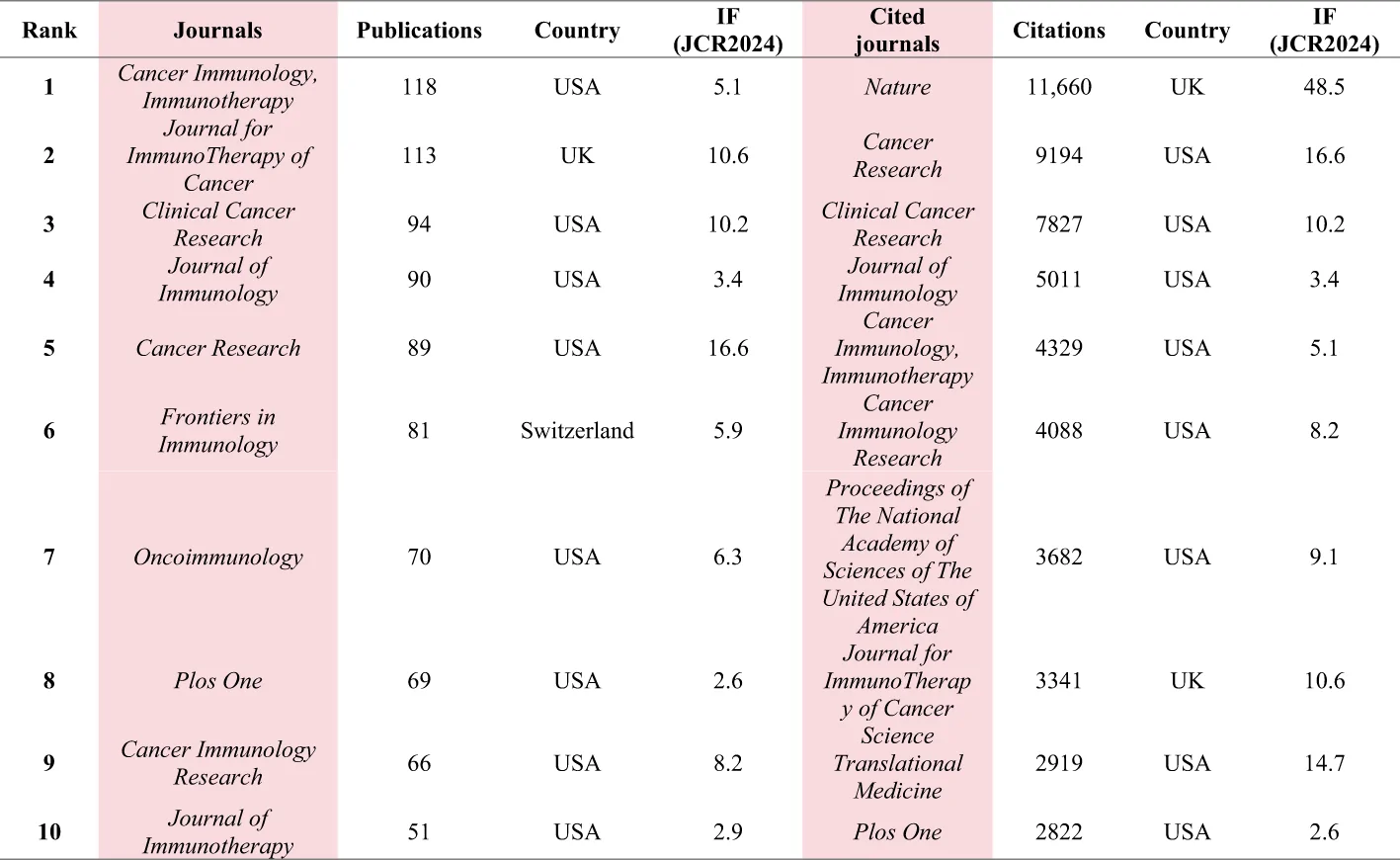
Top 10 journals by publication volume and total citations

### Keyword analysis

**Fig. 14:**
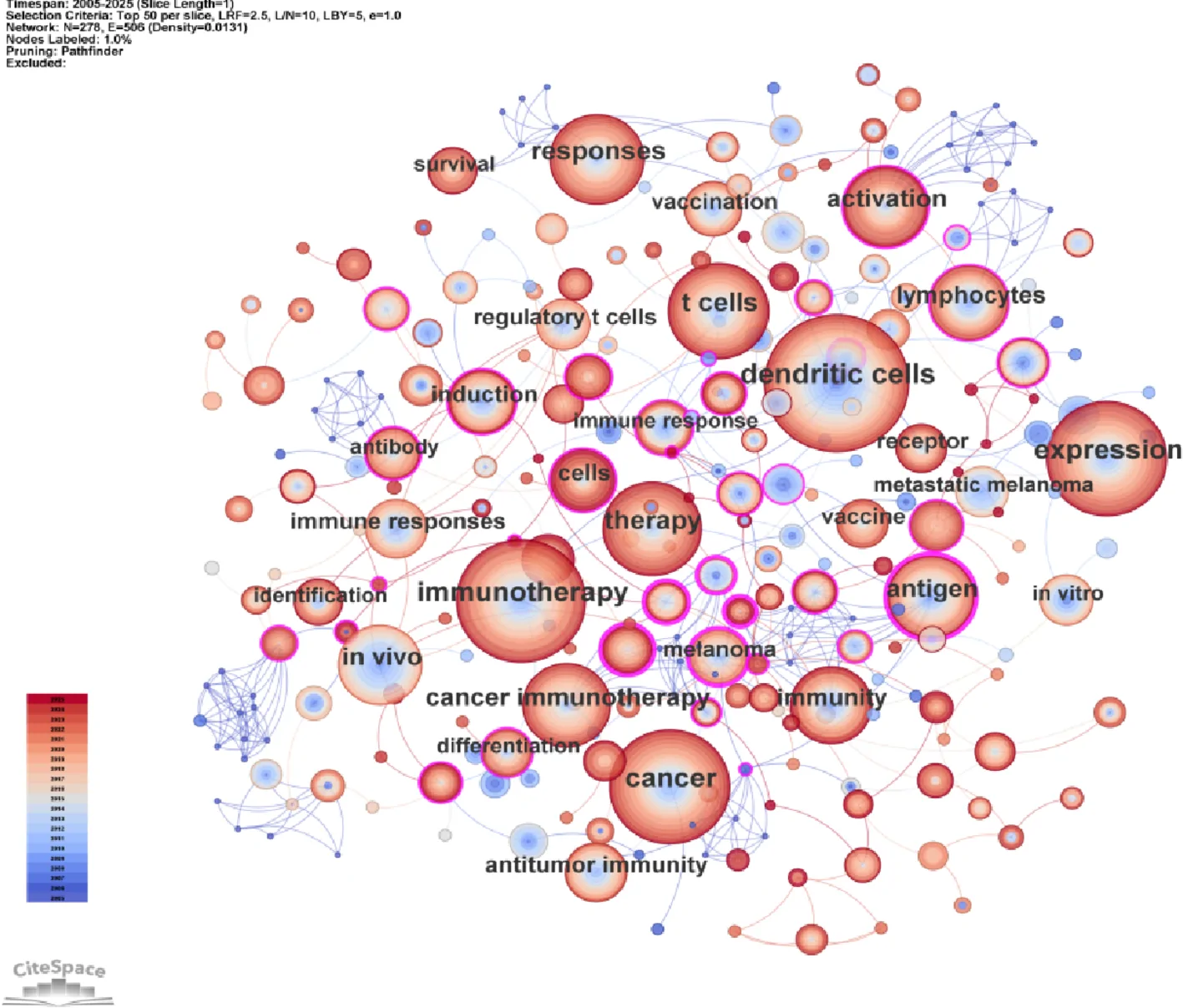
Keyword co-occurrence map

**Fig. 15:**
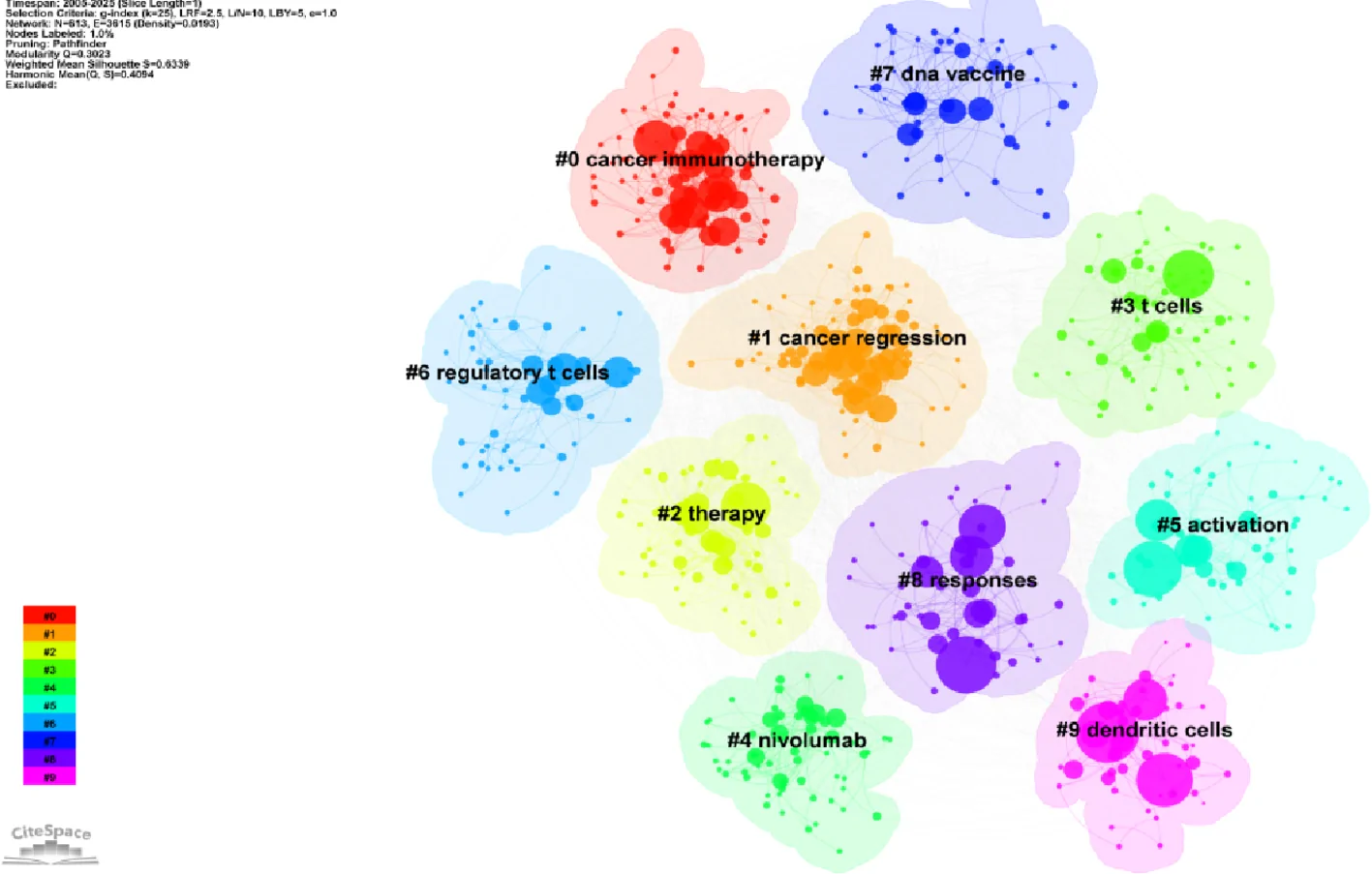
Cluster map of keywords

As shown in Figs. 14 and 15, keywords in the field of tumor antigen-based cancer immunotherapy were statistically analyzed and clustered. “Dendritic cells” was the most frequently mentioned keyword in studies of tumor antigen-specific immunotherapy. It was closely associated with numerous related terms and formed an independent keyword cluster, highlighting the central role of dendritic cells as antigen recipients and presenters in antigen-driven immunotherapy research. In addition to dendritic cells, T cell populations also played important roles in this field. Keywords such as “T cells” and “regulatory T cells” indicate that immune cell-mediated cytotoxicity and immune suppression are key factors influencing the final outcome of antitumor immune responses.

In the keyword clustering analysis, several cluster labels were associated with therapeutic strategies and immune response outcomes. Clusters labeled as “vaccine” and “DNA vaccine” suggest that tumor antigen-based active immune induction constitutes an important research module in antigen-related cancer immunotherapy. In addition to vaccine-related themes, clusters associated with monoclonal antibodies or immune checkpoint-related terms, such as “nivolumab”, indicate that immune checkpoint regulation has also become a major thematic direction within this field. Although immune checkpoint inhibitors do not directly target tumor antigens, they may improve therapeutic efficacy by restoring or enhancing antigen-specific T-cell responses. Moreover, clusters labeled as “cancer regression”, “activation”, and “responses” represent outcome-oriented and mechanism-oriented research themes, reflecting the field’s focus on immune activation, effective antitumor responses, and tumor control.

**Fig. 16:**
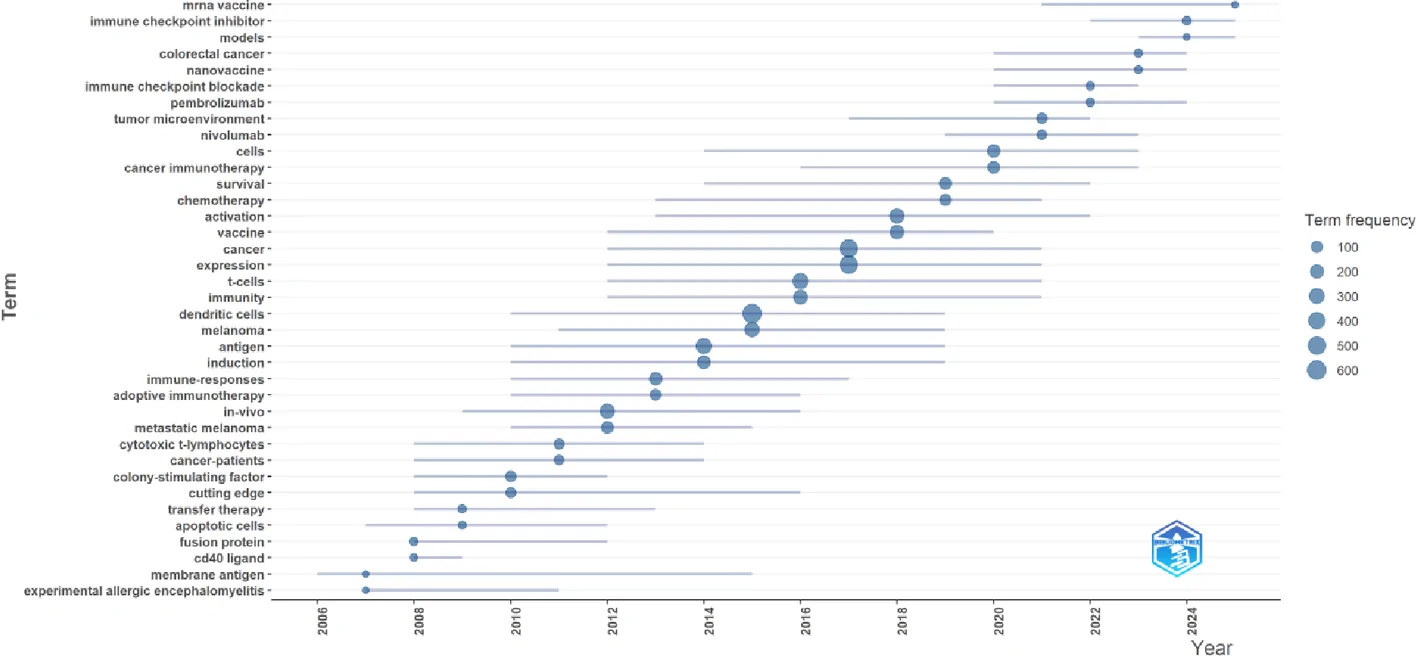
Keyword trend map

Figure 16 shows the high-frequency keywords appearing in different years. The size of each node represents the frequency of keyword occurrence, whereas the length of the line segment on each node indicates the duration of frequent occurrence. In the early stage of research, keywords such as “experimental allergic encephalomyelitis”, “membrane antigen”, and “transfer therapy” mainly reflected studies on antigen recognition, immune response mechanisms, and early explorations of immune cell therapy. With the development of immunological theory and technology, the research focus gradually shifted toward more precise antigen recognition and immune regulation. The emergence of more specific terms such as “cytotoxic T lymphocytes”, “adoptive immunotherapy”, “dendritic cells”, and “tumor microenvironment” indicates that researchers began to examine in greater depth the cellular basis of antigen-specific immune responses and their regulatory mechanisms within the tumor environment.

Since 2021, the appearance of “nivolumab”, “pembrolizumab”, and “immune checkpoint inhibitor” as high-frequency keywords indicates that, after more than a decade of technical accumulation, immune checkpoint inhibitors have developed into important strategies for enhancing antigen-specific antitumor immunity. Moreover, following the successful clinical application of mRNA vaccine platforms in recent years, tumor antigen vaccine formulations have attracted renewed attention. The frequent emergence of “nanovaccine” and “mRNA vaccine” suggests that research on non-protein tumor vaccines has entered a new stage [40, 41].

Overall, the development of tumor antigen-based cancer immunotherapy reflects a progression from the exploration of antigen recognition mechanisms, to the regulation of immune cell function, and further to precise antigen-targeted therapy. With continuous advances in tumor antigen research, immune regulation technologies, and vaccine platforms, this field has gradually formed an integrated research system centered on antigen recognition and connecting basic immunological research with clinical therapeutic translation [42–44].

### Co-citation analysis

Figure 4 presents, from left to right, highly cited publications in the field of tumor antigen-based cancer immunotherapy from 2005 to 2025 and their connections. The top 25 articles by co-citation frequency are listed in Table 5. The study on adoptive T lymphocyte therapy published by Dudley ME and colleagues in 2005 laid an important foundation for the subsequent development of antigen-targeted cellular therapy [45]. Between 2005 and 2010, adoptive therapy using autologous immune cells became one of the major research directions in tumor antigen-based immunotherapy. Rosenberg SA and colleagues systematically reviewed this research trend during that period [46]. Subsequently, the work of Kantoff PW and colleagues on dendritic cell-based therapy further expanded this direction. These early explorations not only advanced the understanding of interactions between immune cells and tumors but also provided a theoretical basis for the later development of antigen-driven immunotherapeutic strategies [47].

**Fig. 17:**
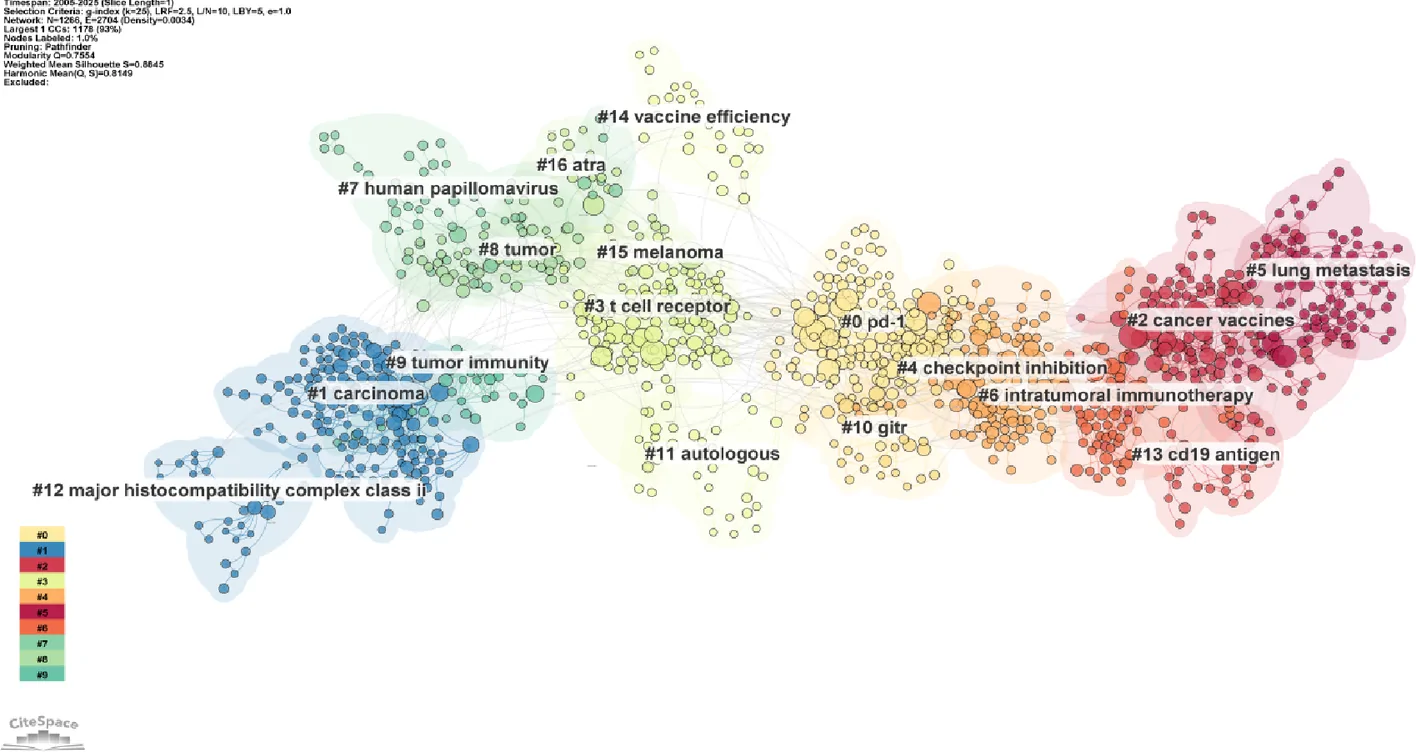
Clustering of literature co-citations

After years of research accumulation, studies on immunosuppression began to expand rapidly around 2010. In 2009, Gabrilovich DI and colleagues summarized and classified myeloid-derived suppressor cells (MDSCs) in *Nature Reviews Immunology*, reflecting the initial clarification of immunosuppressive cells and related mechanisms at that time [48]. In 2010, Hodi FS and colleagues reported positive phase III clinical trial results for ipilimumab, initiating a wave of immune checkpoint research that has continued to the present [15]. Thereafter, as shown in Figs. 4 and 17, immune checkpoint pathways such as PD-1/PD-L1 became major research hotspots. Although immune checkpoint inhibitors do not directly target tumor antigens, their primary mechanism is to relieve immunosuppression and restore the ability of T cells to recognize and kill tumor antigen-bearing cells. Therefore, they have become an important regulatory strategy in tumor antigen-based cancer immunotherapy. Between 2010 and 2015, in addition to the rapid progress in immune checkpoint inhibition, studies by Robbins PF, Palucka K, and others in the co-citation network also reflected the continued exploration of antigen-specific immune cell therapy. These studies further promoted the development of engineered cellular therapies based on artificial antigen recognition, such as CAR-T and TCR-T cell therapies [49, 50].

With the continued deepening of immunological theory and the constant updating of research methods, tumor antigen-based cancer immunotherapy gradually entered a stage of precision-oriented exploration. Scholars such as Schumacher TN and Ott PA pointed out that the widespread application of massively parallel sequencing and machine learning in recent years has enabled researchers to detect noncanonical mutations in tumors on a broader scale, thereby identifying and even rationally designing neoantigens. These advances have provided an important basis for the design of personalized immunotherapeutic strategies. Such breakthroughs in antigen recognition and antigen design have significantly accelerated the movement of cancer immunotherapy toward greater precision [51, 52].

To further verify the evolution of research hotspots in recent years and reduce the bias introduced by the influence of publication time on cumulative citation counts, this study further analyzed the average annual citation frequency of co-cited references (Table 6). Compared with the long-term influential publications identified based on cumulative co-citation frequency in Table 5, average annual citation frequency can more sensitively reflect rapidly developing research directions in recent years. The results showed that approximately 52% of the highly cited publications by average annual citation frequency in Table 6 also appeared in Table 5. These publications mainly included classic studies with long-term influence in immune checkpoint blockade, adoptive cell therapy, and basic tumor immunology. The remaining 48% of publications were mainly published after 2017, reflecting the continuous emergence of new research directions in tumor antigen-based cancer immunotherapy in recent years.

Further analysis of these highly influential recent publications showed that most of them focused on tumor vaccine-related research, including neoantigen identification, personalized vaccine design, and the development of mRNA vaccine technologies [53–57]. In addition, several studies focused on mechanisms of resistance to immunotherapy, including T cell exhaustion and immune escape [58, 59]. These findings indicate that, as immune checkpoint therapy and cellular therapy have gradually entered clinical application, the research focus has further expanded toward the precise identification of tumor-specific antigens and the optimization of immune response regulation.

As described by Saxena M and colleagues, and as further supported by the document co-citation cluster map shown in Fig. 17, in-depth research on tumor antigens and their recognition and delivery mechanisms has laid a critical foundation for the development of tumor vaccines and has contributed to the renewed vitality of this field in recent years. The successful development and clinical application of HPV vaccines have further demonstrated the feasibility of actively activating the immune system to prevent and control tumors. However, compared with traditional prophylactic vaccines, therapeutic tumor vaccines still face challenges related to antigen selection, the strength of immune responses, and the efficiency of clinical translation. In the future, with continued advances in antigen prediction, delivery technologies, and immune regulatory strategies, tumor antigen-based precision immunotherapy is expected to retain broad developmental potential [60].

**Table 5:**
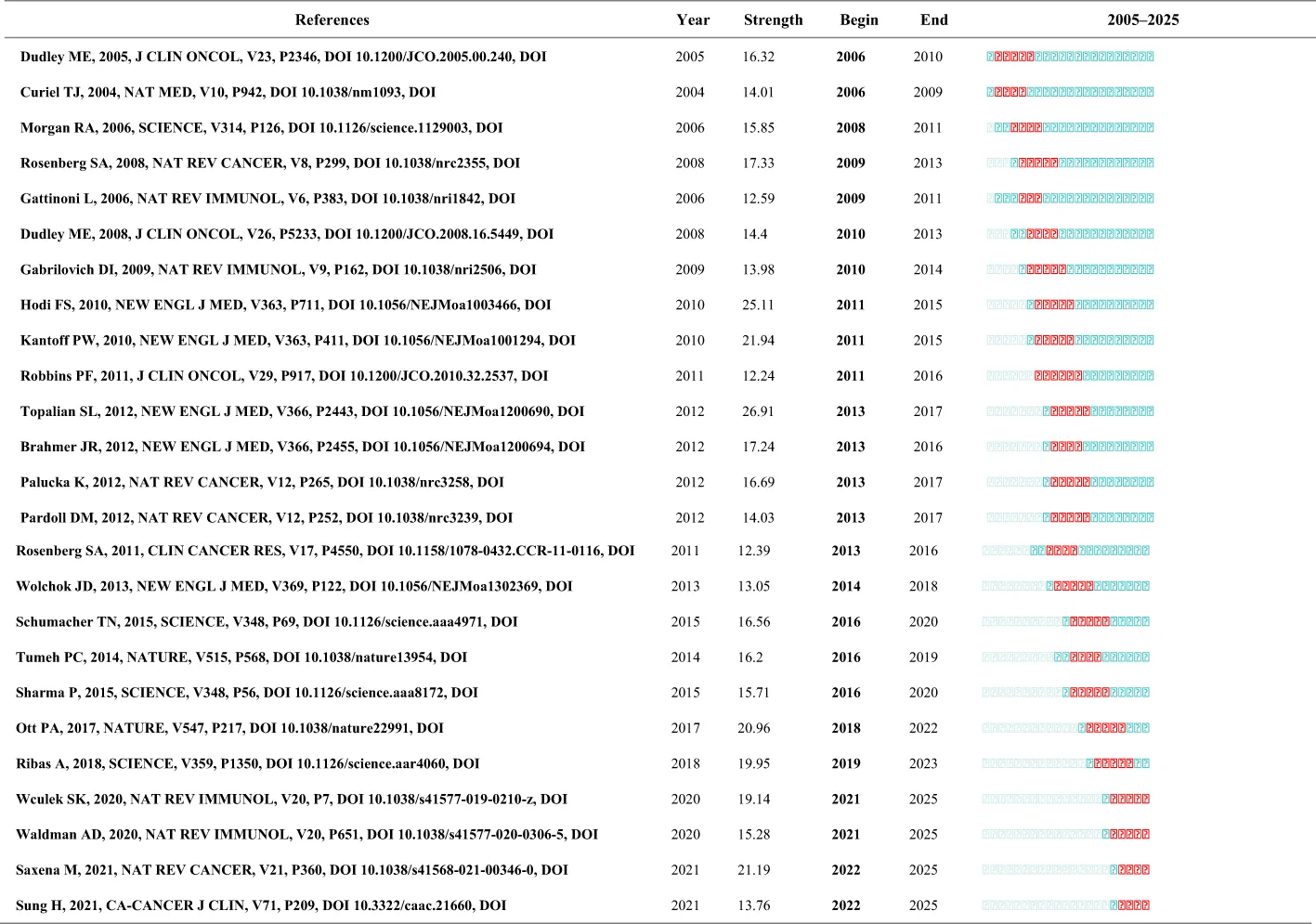
Top 25 highly co-cited references

**Table 6:** Top 25 References by average annual co-citation frequency

References	Year	Citation count	Citation ranking	Annual average citations
Saxena M, 2021, NAT REV CANCER, V21, P360, DOI https://doi.org/10.1038/s41568-021-00346-0	2021	44	6	11.87
Waldman AD, 2020, NAT REV IMMUNOL, V20, P651, DOI https://doi.org/10.1038/s41577-020-0306-5	2020	36	11	7.65
Fan T, 2023, SIGNAL TRANSDUCT TAR, V8, P0, DOI https://doi.org/10.1038/s41392-023-01674-3	2023	12	87	7.02
Rojas LA, 2023, NATURE, V618, P144, DOI https://doi.org/10.1038/s41586-023-06063-y	2023	12	92	7.02
Sung H, 2021, CA-CANCER J CLIN, V71, P209, DOI https://doi.org/10.3322/caac.21660	2021	26	27	7.01
Ribas A, 2018, SCIENCE, V359, P1350, DOI https://doi.org/10.1126/science.aar4060	2018	46	5	6.86
Ott PA, 2017, NATURE, V547, P217, DOI https://doi.org/10.1038/nature22991	2017	52	3	6.75
Lin MJ, 2022, NAT CANCER, V3, P911, DOI https://doi.org/10.1038/s43018-022-00418-6	2022	16	63	5.91
Wculek SK, 2020, NAT REV IMMUNOL, V20, P7, DOI https://doi.org/10.1038/s41577-019-0210-z	2020	27	21	5.73
Blass E, 2021, NAT REV CLIN ONCOL, V18, P215, DOI https://doi.org/10.1038/s41571-020-00460-2	2021	19	46	5.12
Topalian SL, 2012, NEW ENGL J MED, V366, P2443, DOI https://doi.org/10.1056/NEJMoa1200690	2012	61	1	4.80
Sahin U, 2020, NATURE, V585, P107, DOI https://doi.org/10.1038/s41586-020-2537-9	2020	22	38	4.67
Schumacher TN, 2015, SCIENCE, V348, P69, DOI https://doi.org/10.1126/science.aaa4971	2015	39	7	4.02
Sahin U, 2017, NATURE, V547, P222, DOI https://doi.org/10.1038/nature23003	2017	30	17	3.89
Hodi FS, 2010, NEW ENGL J MED, V363, P711, DOI https://doi.org/10.1056/NEJMoa1003466	2010	56	2	3.81
Miller BC, 2019, NAT IMMUNOL, V20, P326, DOI https://doi.org/10.1038/s41590-019-0312-6	2019	21	39	3.68
Sharma P, 2015, SCIENCE, V348, P56, DOI https://doi.org/10.1126/science.aaa8172	2015	34	12	3.50
Kantoff PW, 2010, NEW ENGL J MED, V363, P411, DOI https://doi.org/10.1056/NEJMoa1001294	2010	49	4	3.33
Hilf N, 2019, NATURE, V565, P240, DOI https://doi.org/10.1038/s41586-018-0810-y	2019	19	44	3.33
Lorentzen CL, 2022, LANCET ONCOL, V23, PE450, DOI https://doi.org/10.1016/S1470-2045(22)00372-2	2022	9	126	3.32
Sharma P, 2017, CELL, V168, P707, DOI https://doi.org/10.1016/j.cell.2017.01.017	2017	25	29	3.24
Keskin DB, 2019, NATURE, V565, P234, DOI https://doi.org/10.1038/s41586-018-0792-9	2019	18	48	3.15
Spranger S, 2017, CANCER CELL, V31, P711, DOI https://doi.org/10.1016/j.ccell.2017.04.003	2017	24	31	3.11
Tumeh PC, 2014, NATURE, V515, P568, DOI https://doi.org/10.1038/nature13954	2014	33	14	3.08
Palucka K, 2012, NAT REV CANCER, V12, P265, DOI https://doi.org/10.1038/nrc3258	2012	38	8	2.99

## Discussion

### Overview

Since 2005, tumor antigen-based cancer immunotherapy has evolved from the exploration of antigen recognition mechanisms, to immune cell-targeted therapy, and further to the development of personalized vaccines. It has gradually become an important direction in precision immunotherapy. Multiple countries have played essential roles in this research area. The United States has successfully extended its long-standing leadership in immunology, oncology, and related fields to this interdisciplinary area of tumor antigen-based cancer immunotherapy. It has remained relatively leading in terms of publication output, research quality, and frontier contributions. Germany, the United Kingdom, Japan, and other countries have maintained positions in the second tier of global contributors, supported by mature research systems and international collaboration networks. China has shown rapid growth in publication output in recent years, reflecting important progress in biomedical research investment, talent training, and research infrastructure development. However, compared with some traditional research powers in Europe and North America, the average citation level of Chinese publications remains relatively lower, which may be related to factors such as international collaboration patterns, journal influence, and the duration of academic accumulation.

From the perspective of institutions and research team distribution, biomedical research clusters on the East Coast of the United States occupy an important position in tumor antigen-based cancer immunotherapy research. For example, Professor Chien-Fu Hung and his team at Johns Hopkins University have been conducting research on tumor vaccines, vaccine adjuvants, and immune mechanisms since the early formation of this field. By 2025, they continued to contribute to the research frontier and had become one of the highly influential teams in this area. Similarly, Professor Jeffrey Schlom and his team at the NCI have long been engaged in research on tumor antigens, cancer vaccines, and immunotherapy, making substantial contributions to the development of the field. In addition to these long-standing academic teams, emerging researchers such as Ugur Sahin, Wei Zhang, and Jun-O Jin have published several high-quality studies in recent years and have become increasingly influential figures in both academic research and translational development.

### Developmental trajectory and driving forces

The development of tumor antigen-based cancer immunotherapy is essentially the result of the integration of immunology, tumor biology, and translational medicine. Its breakthroughs have depended on the elucidation of key immune mechanisms and the development of related technological platforms. Between 2005 and 2010, the theories of cancer immunosurveillance and immunoediting were gradually refined, while the mechanisms of immune checkpoint pathways, including CTLA-4 and PD-1, were increasingly clarified. These advances laid an important theoretical foundation for subsequent immune checkpoint blockade therapy.

Subsequently, the clinical success of immune checkpoint inhibitors promoted the development of antigen-related immune regulatory strategies. Therapeutic approaches represented by the CTLA-4 antibody ipilimumab and PD-1/PD-L1 antibodies restore the ability of T cells to recognize and kill tumor antigen-bearing cells by relieving immunosuppression. However, with the expanding clinical application of these therapies, researchers gradually recognized that immune escape and tumor microenvironment-mediated immunosuppression are important factors limiting therapeutic efficacy [61, 62]. In response to resistance to immune checkpoint therapy, researchers have further explored novel immune regulatory targets, such as LAG-3, TIGIT, and TIM-3, with the aim of improving therapeutic responses through multi-pathway combinatorial regulation [63].

At the same time, advances in cellular engineering technologies have further promoted the development of adoptive immunotherapy. The successful approval of CAR-T cell therapy marked the entry of engineered immune cell therapy based on artificial antigen recognition modules into clinical application, while tumor-infiltrating lymphocyte therapy and TCR-engineered T cell therapy have further expanded therapeutic strategies targeting solid tumors and specific antigen recognition. Among these approaches, TCR-T cells can recognize intracellular antigens presented by MHC molecules, providing a new direction for the treatment of solid tumors that are difficult to cover with conventional CAR-T therapy. In addition, emerging cellular therapy platforms, such as CAR-NK cells, have gradually attracted attention and offer new possibilities for next-generation cellular immunotherapy [64, 65].

In recent years, advances in high-throughput sequencing, computational prediction, and mRNA technologies have further promoted neoantigen discovery and the development of personalized cancer vaccines, enabling researchers to design more precise antigen-specific immunotherapeutic strategies based on patient-specific mutations. By identifying patient-specific mutations and designing targeted immune strategies, personalized vaccines have gradually become an important direction in precision immunotherapy. Meanwhile, bispecific antibodies enhance interactions between immune cells and tumor cells, providing a new strategy for overcoming the limitations of traditional therapeutic models [66, 67].

Overall, the development of cancer immunotherapy has not been driven by a single technology. Rather, it is the result of the combined effects of basic immune mechanism elucidation, clinical needs, and advances in engineering technologies.

### Unresolved challenges and future research directions

Although tumor antigen-based cancer immunotherapy has made substantial progress, research growth in this field has gradually stabilized in recent years, suggesting that current therapeutic models still face a series of unresolved key challenges.

Resistance to immunotherapy remains one of the major barriers limiting clinical benefit. Some patients develop primary or acquired resistance to immune checkpoint inhibitors due to tumor antigen loss, abnormal antigen presentation, and activation of immune escape mechanisms. In addition, T cell exhaustion induced by long-term antigen stimulation and the formation of an immunosuppressive tumor microenvironment can also weaken the ability of immune cells to exert sustained antitumor effects [68, 69].

At the same time, the complex biological characteristics of solid tumors further limit the application of cellular therapy strategies. Although CAR-T cell therapy has achieved major breakthroughs in hematological malignancies, its efficacy in solid tumors remains limited by antigen heterogeneity, immunosuppressive barriers, and insufficient infiltration of effector cells. Therefore, future studies should further improve the tumor recognition capacity and sustained cytotoxic activity of immune cells by integrating strategies such as TCR-T therapy, optimized TIL therapy, and bispecific antibodies [70, 71].

For personalized immunotherapy, neoantigen vaccines have shown promising application prospects, but their further clinical translation remains constrained by factors such as the accuracy of antigen prediction, manufacturing timelines, and individual differences in patient immune status. In the future, the integration of artificial intelligence, multi-omics analysis, and high-throughput screening technologies is expected to improve patient selection efficiency and optimize the design of personalized therapeutic strategies [56].

In addition, establishing more reliable systems for predicting therapeutic efficacy is also essential for promoting the precision development of immunotherapy. At present, indicators such as PD-L1 expression, tumor mutational burden, and immune cell infiltration status remain insufficient to fully predict patient responses to treatment. Future research should integrate multidimensional biological information to achieve more accurate patient stratification and therapeutic efficacy assessment [72].

Overall, the future development of cancer immunotherapy will depend not only on the discovery of new therapeutic targets, but also on a deeper understanding of the complex and dynamic interactions between tumors and the immune system. Through the rational combination of multiple therapeutic strategies, more durable and precise antitumor immunity may ultimately be achieved.

### Limitations

Although we attempted to conduct a practical and comprehensive analysis of the literature on tumor antigen-based cancer immunotherapy, this study still has several limitations. First, only the Web of Science Core Collection (WoSCC) was selected as the retrieval database. Although this approach reduced potential duplication and inconsistencies caused by merging multiple databases, it also limited the breadth of the literature covered in this study to some extent. In addition, because only English-language publications were included, language bias may exist, and some relevant studies published in non-English languages may not have been included. Although English-language publications usually constitute a major component of international academic communication, this restriction may still have affected the comprehensiveness of the results.

Second, there is usually a time lag between the conduct of research and its publication. Therefore, this study mainly reflects developmental trends in the field over a past period and may not fully represent the latest ongoing research. Moreover, because the search was conducted on September 15, 2025, the publication data for 2025 covered only the first 8.5 months of that year and therefore represented incomplete annual data. The estimation of the number of publications for 2025 based on previous growth trends was used only as a trend reference, and the related results should be further verified using complete annual data.

Third, bibliometric analysis relies on quantitative indicators such as publication output, citation counts, and network structure, which may be influenced by citation practices, disciplinary differences, and time accumulation effects. For example, cumulative citation counts tend to reflect the long-term influence of earlier publications and may underestimate recent high-impact studies. Review articles also usually accumulate relatively high citation counts, and their inclusion or exclusion may affect document co-citation relationships and knowledge structure analyses. To assess this potential influence, this study further conducted a sensitivity analysis comparing datasets that included review articles with datasets that included only research articles. The relevant results are provided in the Supplementary Materials. The results showed that although the inclusion of review articles affected some keyword frequencies and network connections to a certain extent, the two datasets remained highly consistent in terms of major research themes, keyword evolution trends, core co-cited references, and clustering structures. Both datasets identified key research directions, including immune checkpoint therapy, adoptive cell therapy, tumor antigen recognition, and personalized neoantigen vaccines. Therefore, excluding review articles did not substantially alter the overall interpretation of this study regarding the developmental trajectory of tumor immunotherapy.

Moreover, beyond the limitations associated with quantitative indicators, interpretations of bibliometric findings may involve a degree of subjectivity. Although these analyses provide insights into research evolution, the interpretation of publication trends, keyword patterns, and network structures may be influenced by analytical perspectives and should therefore be considered with caution.

Finally, this study used a keyword-based screening strategy that included antigen-related terms. Although this strategy helped focus the analysis on research directions involving antigen recognition, antigen-specific immune responses, and antigen-targeted therapy, it may have missed some immunotherapy studies that did not explicitly use these terms, such as certain studies related to immune checkpoint inhibitors, CAR-T cell therapy, and the tumor microenvironment. Therefore, the results of this study mainly reflect the developmental trends in tumor antigen-based cancer immunotherapy and cannot fully represent all research directions within the broader field of cancer immunotherapy.

## Conclusion

This study analyzed the developmental trajectory of tumor antigen-based cancer immunotherapy over the past two decades. In the context of broad global participation, the United States has led the research frontier and collaboration network in this field, while emerging countries such as China have gradually become important contributors to academic output. Institutions such as Johns Hopkins University and the National Cancer Institute have conducted long-term research on tumor antigen recognition, immune regulation, and vaccine development, continuously promoting the clinical and industrial translation of related research.

The results indicate that tumor antigen-based cancer immunotherapy has evolved from early adoptive immune cell therapy and the exploration of antigen presentation mechanisms to immune checkpoint regulation, engineered antigen-recognition cell therapy, and personalized neoantigen vaccine development. With the advancement of emerging technologies such as high-throughput sequencing, artificial intelligence-based prediction, and mRNA technology, the research focus has gradually shifted from simply enhancing immune activation to precisely identifying tumor-specific antigens and optimizing antigen-driven immune responses. In the future, the integration of antigen prediction, delivery system optimization, and multimodal immune combination strategies is expected to further promote the development of cancer immunotherapy toward greater precision, efficacy, and personalization.

## Supplementary Information

Below is the link to the electronic supplementary material.

Supplementary Material 1.

## Data Availability

The datasets generated and/or analyzed during the current study are available from the corresponding author on reasonable request. Bibliographic data used in this study were retrieved from the Web of Science Core Collection database.
