# Supplementary material for: The evolution of antigen specific cancer immunotherapy from adoptive cell therapy to personalized cancer vaccines
Source: Discov Oncol. 2026 Aug 5;17:1511. doi: 10.1007/s12672-026-05719-1 (PMC13727929; doi:10.1007/s12672-026-05719-1)
Supplement: Supplementary file 1 [file 12672_2026_5719_MOESM1_ESM.docx]

**Sensitivity analysis of the influence of review article inclusion**

**on research themes and knowledge structure**

To evaluate the potential impact of excluding review articles on bibliometric findings, sensitivity analyses were performed by comparing datasets containing both research articles and review articles with datasets containing research articles only. The influence of review article inclusion was assessed using keyword analysis, keyword burst detection, keyword clustering, co-citation analysis, citation burst analysis, and co-citation clustering.

Due to the dynamic updating nature of the Web of Science database, the original dataset containing both research articles and review articles was not retained during the initial analysis, while the dataset containing research articles only was preserved. Therefore, the present sensitivity analysis was conducted using the currently retrieved datasets based on the same search strategy. Compared with the previously obtained dataset, the updated dataset contained a slight increase in the number of records, including four additional research articles and three additional review articles. These differences resulted from continuous database updates rather than changes in the retrieval strategy.

Because bibliometric visualization involves clustering algorithms and network layout optimization, minor differences in node distribution and graphical presentation may occur between analyses based on different database versions. However, these variations do not alter the fundamental interpretation of the results, as the major research themes, knowledge structures, and evolutionary trends identified from the updated datasets remained highly consistent. Therefore, although the newly generated visualizations may not be completely identical to those obtained from the original database version, the comparison between the datasets with and without review articles at the same retrieval time point provides reliable evidence that the exclusion of review articles has limited influence on the overall bibliometric interpretation of cancer immunotherapy research.

The selection of these indicators was based on their ability to directly reflect the knowledge structure and research evolution of a scientific field. Keyword analyses provide information regarding major research topics, emerging trends, and temporal shifts in scientific interests, whereas co-citation analyses reveal the intellectual foundations, influential studies, and evolutionary pathways of the field. Since review articles mainly function as knowledge integration and synthesis documents, their inclusion is more likely to influence conceptual structures and citation relationships rather than the distribution of research contributors. Therefore, analyses focusing on countries, institutions, authors, and journals were not included in this sensitivity analysis, as these indicators primarily reflect patterns of scientific productivity and collaboration, which are less directly affected by the inclusion or exclusion of review articles.

For better visualization, differences between the two datasets are highlighted in blue in the corresponding tables.

**Influence of review article inclusion on keyword analysis**

The inclusion of review articles slightly increased the frequency of several keywords, reflecting the broader conceptual summarization characteristics of review publications. However, the main research topics remained highly consistent between the two datasets. As shown in Table S1, keywords with the highest occurrence frequencies in both datasets were mainly associated with fundamental themes of cancer immunotherapy, including dendritic cells, immunotherapy, cancer, T cells, antigen recognition, immune responses, and tumor microenvironment. Although some differences existed in the ranking and frequency of individual keywords, no major differences were observed in the overall research focus.

Keyword burst analysis further demonstrated similar temporal evolution patterns between datasets (Table S2). Early research hotspots in both datasets included tumor immunity, cytotoxic T lymphocytes, tumor antigens, and adoptive immunotherapy, reflecting the initial exploration of immune-based cancer treatment. In later periods, both datasets identified immune checkpoint blockade, nivolumab, resistance, tumor microenvironment, and immune checkpoint inhibitors as emerging research directions, indicating that the exclusion of review articles did not substantially influence the identification of major research transitions.

**Influence of review article inclusion on keyword clustering**

Keyword clustering analysis showed that the overall knowledge structure remained comparable after excluding review articles (Table S3 and Figures S1–S2). Both datasets identified major research clusters related to cancer immunotherapy, antigen presentation, dendritic cells, immune checkpoint therapy, tumor microenvironment, vaccines, and adoptive immunotherapy.

Although cluster labels and the number of keywords within individual clusters varied slightly between datasets, these differences mainly reflected the broader conceptual integration characteristics of review articles. The core biological themes and evolutionary directions remained unchanged, suggesting that review article exclusion did not significantly distort the interpretation of research hotspots.

Table S1. Top 50 Most Frequently Occurring Keywords in Datasets With and Without Review Articles

| **Research and Review** | | | | | | **Research** | | | | | |
| --- | --- | --- | --- | --- | --- | --- | --- | --- | --- | --- | --- |
| **Rank** | **Keywords** | **count** | **Rank** | **Keywords** | **count** | **Rank** | **Keywords** | **count** | **Rank** | **Keywords** | **count** |
| 1 | dendritic cells | 769 | 26 | identification | 138 | 1 | dendritic cells | 604 | 26 | immune response | 116 |
| 2 | immunotherapy | 584 | 27 | antibody | 136 | 2 | immunotherapy | 517 | 27 | survival | 116 |
| 3 | expression | 568 | 28 | survival | 134 | 3 | expression | 514 | 28 | delivery | 114 |
| 4 | cancer | 503 | 29 | breast cancer | 133 | 4 | cancer | 462 | 29 | differentiation | 114 |
| 5 | t cells | 486 | 30 | vaccine | 133 | 5 | t cells | 389 | 30 | blockade | 110 |
| 6 | therapy | 408 | 31 | blockade | 124 | 6 | therapy | 366 | 31 | in vitro | 109 |
| 7 | responses | 364 | 32 | differentiation | 121 | 7 | responses | 333 | 32 | vaccines | 108 |
| 8 | cancer immunotherapy | 320 | 33 | delivery | 121 | 8 | antigen | 285 | 33 | breast cancer | 107 |
| 9 | antigen | 317 | 34 | adoptive immunotherapy | 119 | 9 | activation | 272 | 34 | cd8(+) t cells | 105 |
| 10 | in vivo | 315 | 35 | tumor microenvironment | 119 | 10 | cancer immunotherapy | 271 | 35 | tumor | 99 |
| 11 | activation | 310 | 36 | tumor | 111 | 11 | in vivo | 262 | 36 | mice | 95 |
| 12 | lymphocytes | 241 | 37 | vaccines | 111 | 12 | immunity | 227 | 37 | combination | 89 |
| 13 | immunity | 240 | 38 | monoclonal antibody | 108 | 13 | lymphocytes | 213 | 38 | protein | 87 |
| 14 | immune responses | 230 | 39 | colony stimulating factor | 106 | 14 | induction | 178 | 39 | chemotherapy | 86 |
| 15 | regulatory t cells | 223 | 40 | t cell responses | 102 | 15 | immune responses | 175 | 40 | generation | 84 |
| 16 | antitumor immunity | 218 | 41 | tumor infiltrating lymphocytes | 101 | 16 | antitumor immunity | 161 | 41 | efficacy | 84 |
| 17 | induction | 196 | 42 | combination | 100 | 17 | cells | 161 | 42 | immunization | 81 |
| 18 | metastatic melanoma | 184 | 43 | mice | 100 | 18 | melanoma | 157 | 43 | tumor microenvironment | 80 |
| 19 | melanoma | 175 | 44 | protein | 100 | 19 | regulatory t cells | 145 | 44 | cancer vaccine | 79 |
| 20 | cells | 175 | 45 | efficacy | 99 | 20 | vaccination | 142 | 45 | suppressor cells | 75 |
| 21 | receptor | 167 | 46 | chemotherapy | 99 | 21 | receptor | 134 | 46 | cytotoxic t lymphocytes | 75 |
| 22 | vaccination | 160 | 47 | dendritic cell | 98 | 22 | identification | 124 | 47 | adoptive immunotherapy | 74 |
| 23 | immune response | 152 | 48 | cancer vaccine | 98 | 23 | metastatic melanoma | 122 | 48 | carcinoma | 73 |
| 24 | in vitro | 146 | 49 | suppressor cells | 98 | 24 | vaccine | 122 | 49 | ifn gamma | 72 |
| 25 | cd8(+) t cells | 140 | 50 | antitumor activity | 95 | 25 | antibody | 117 | 50 | dendritic cell | 71 |

Table S2. Top 25 Keywords with the Strongest Citation Bursts in Datasets With and Without Review Articles

| **Research and Review** | | | | | | **Research** | | | | | |
| --- | --- | --- | --- | --- | --- | --- | --- | --- | --- | --- | --- |
| **Keywords** | **Year** | **Strength** | **Begin** | **End** | **2005 - 2025** | **Keywords** | **Year** | **Strength** | **Begin** | **End** | **2005 - 2025** |
| colony stimulating factor | 2005 | 25.37 | **2005** | 2010 | ▃▃▃▃▃▃▂▂▂▂▂▂▂▂▂▂▂▂▂▂▂ | in vivo | 2005 | 17.97 | **2005** | 2013 | ▃▃▃▃▃▃▃▃▃▂▂▂▂▂▂▂▂▂▂▂▂ |
| in vivo | 2005 | 22.83 | **2005** | 2013 | ▃▃▃▃▃▃▃▃▃▂▂▂▂▂▂▂▂▂▂▂▂ | tumor necrosis factor | 2005 | 9.17 | **2005** | 2013 | ▃▃▃▃▃▃▃▃▃▂▂▂▂▂▂▂▂▂▂▂▂ |
| tumor necrosis factor | 2005 | 12.62 | **2005** | 2013 | ▃▃▃▃▃▃▃▃▃▂▂▂▂▂▂▂▂▂▂▂▂ | colony stimulating factor | 2006 | 18.8 | **2006** | 2012 | ▂▃▃▃▃▃▃▃▂▂▂▂▂▂▂▂▂▂▂▂▂ |
| tumor immunity | 2006 | 17.76 | **2006** | 2013 | ▂▃▃▃▃▃▃▃▃▂▂▂▂▂▂▂▂▂▂▂▂ | tumor immunity | 2006 | 16.47 | **2006** | 2013 | ▂▃▃▃▃▃▃▃▃▂▂▂▂▂▂▂▂▂▂▂▂ |
| cytotoxic t lymphocytes | 2006 | 12.73 | **2006** | 2012 | ▂▃▃▃▃▃▃▃▂▂▂▂▂▂▂▂▂▂▂▂▂ | gene transfer | 2006 | 8.43 | **2006** | 2011 | ▂▃▃▃▃▃▃▂▂▂▂▂▂▂▂▂▂▂▂▂▂ |
| gene transfer | 2006 | 10.63 | **2006** | 2011 | ▂▃▃▃▃▃▃▂▂▂▂▂▂▂▂▂▂▂▂▂▂ | in vitro | 2006 | 8.1 | **2006** | 2008 | ▂▃▃▃▂▂▂▂▂▂▂▂▂▂▂▂▂▂▂▂▂ |
| prostate cancer | 2006 | 7.93 | **2006** | 2012 | ▂▃▃▃▃▃▃▃▂▂▂▂▂▂▂▂▂▂▂▂▂ | cytotoxic t lymphocytes | 2006 | 7.9 | **2006** | 2008 | ▂▃▃▃▂▂▂▂▂▂▂▂▂▂▂▂▂▂▂▂▂ |
| in vitro | 2006 | 7.81 | **2006** | 2008 | ▂▃▃▃▂▂▂▂▂▂▂▂▂▂▂▂▂▂▂▂▂ | cancer patients | 2007 | 11.47 | **2007** | 2012 | ▂▂▃▃▃▃▃▃▂▂▂▂▂▂▂▂▂▂▂▂▂ |
| cancer patients | 2006 | 12.16 | **2007** | 2012 | ▂▂▃▃▃▃▃▃▂▂▂▂▂▂▂▂▂▂▂▂▂ | cutting edge | 2007 | 9.71 | **2007** | 2010 | ▂▂▃▃▃▃▂▂▂▂▂▂▂▂▂▂▂▂▂▂▂ |
| cutting edge | 2006 | 10.92 | **2007** | 2010 | ▂▂▃▃▃▃▂▂▂▂▂▂▂▂▂▂▂▂▂▂▂ | tumor antigen | 2007 | 7.31 | **2007** | 2012 | ▂▂▃▃▃▃▃▃▂▂▂▂▂▂▂▂▂▂▂▂▂ |
| tumor antigen | 2005 | 7.78 | **2007** | 2012 | ▂▂▃▃▃▃▃▃▂▂▂▂▂▂▂▂▂▂▂▂▂ | metastatic melanoma | 2006 | 11.73 | **2009** | 2013 | ▂▂▂▂▃▃▃▃▃▂▂▂▂▂▂▂▂▂▂▂▂ |
| metastatic melanoma | 2005 | 15.67 | **2009** | 2013 | ▂▂▂▂▃▃▃▃▃▂▂▂▂▂▂▂▂▂▂▂▂ | adoptive immunotherapy | 2006 | 7.43 | **2009** | 2014 | ▂▂▂▂▃▃▃▃▃▃▂▂▂▂▂▂▂▂▂▂▂ |
| multiple myeloma | 2010 | 7.82 | **2012** | 2014 | ▂▂▂▂▂▂▂▃▃▃▂▂▂▂▂▂▂▂▂▂▂ | multiple myeloma | 2010 | 8.18 | **2010** | 2014 | ▂▂▂▂▂▃▃▃▃▃▂▂▂▂▂▂▂▂▂▂▂ |
| nanoparticles | 2017 | 14.79 | **2017** | 2025 | ▂▂▂▂▂▂▂▂▂▂▂▂▃▃▃▃▃▃▃▃▃ | suppressor cells | 2010 | 7.2 | **2012** | 2017 | ▂▂▂▂▂▂▂▃▃▃▃▃▃▂▂▂▂▂▂▂▂ |
| resistance | 2018 | 14.16 | **2018** | 2025 | ▂▂▂▂▂▂▂▂▂▂▂▂▂▃▃▃▃▃▃▃▃ | ovarian cancer | 2008 | 7.93 | **2013** | 2016 | ▂▂▂▂▂▂▂▂▃▃▃▃▂▂▂▂▂▂▂▂▂ |
| checkpoint blockade | 2013 | 11.37 | **2018** | 2022 | ▂▂▂▂▂▂▂▂▂▂▂▂▂▃▃▃▃▃▂▂▂ | nanoparticles | 2017 | 15.69 | **2017** | 2025 | ▂▂▂▂▂▂▂▂▂▂▂▂▃▃▃▃▃▃▃▃▃ |
| nivolumab | 2016 | 11.17 | **2018** | 2025 | ▂▂▂▂▂▂▂▂▂▂▂▂▂▃▃▃▃▃▃▃▃ | checkpoint blockade | 2016 | 9.48 | **2017** | 2021 | ▂▂▂▂▂▂▂▂▂▂▂▂▃▃▃▃▃▂▂▂▂ |
| immune checkpoint blockade | 2018 | 10.13 | **2018** | 2022 | ▂▂▂▂▂▂▂▂▂▂▂▂▂▃▃▃▃▃▂▂▂ | resistance | 2018 | 13.99 | **2018** | 2025 | ▂▂▂▂▂▂▂▂▂▂▂▂▂▃▃▃▃▃▃▃▃ |
| pd 1 blockade | 2016 | 9.17 | **2018** | 2025 | ▂▂▂▂▂▂▂▂▂▂▂▂▂▃▃▃▃▃▃▃▃ | blockade | 2007 | 13.23 | **2018** | 2022 | ▂▂▂▂▂▂▂▂▂▂▂▂▂▃▃▃▃▃▂▂▂ |
| blockade | 2007 | 12.14 | **2019** | 2022 | ▂▂▂▂▂▂▂▂▂▂▂▂▂▂▃▃▃▃▂▂▂ | nivolumab | 2016 | 10.66 | **2018** | 2025 | ▂▂▂▂▂▂▂▂▂▂▂▂▂▃▃▃▃▃▃▃▃ |
| immunogenic cell death | 2019 | 10.57 | **2019** | 2025 | ▂▂▂▂▂▂▂▂▂▂▂▂▂▂▃▃▃▃▃▃▃ | delivery | 2008 | 7.71 | **2018** | 2025 | ▂▂▂▂▂▂▂▂▂▂▂▂▂▃▃▃▃▃▃▃▃ |
| immune checkpoint inhibitors | 2018 | 9.55 | **2020** | 2025 | ▂▂▂▂▂▂▂▂▂▂▂▂▂▂▂▃▃▃▃▃▃ | pd 1 blockade | 2016 | 7.15 | **2018** | 2021 | ▂▂▂▂▂▂▂▂▂▂▂▂▂▃▃▃▃▂▂▂▂ |
| tumor microenvironment | 2015 | 17.61 | **2021** | 2025 | ▂▂▂▂▂▂▂▂▂▂▂▂▂▂▂▂▃▃▃▃▃ | immune checkpoint blockade | 2019 | 8.8 | **2019** | 2025 | ▂▂▂▂▂▂▂▂▂▂▂▂▂▂▃▃▃▃▃▃▃ |
| pembrolizumab | 2018 | 8.09 | **2022** | 2025 | ▂▂▂▂▂▂▂▂▂▂▂▂▂▂▂▂▂▃▃▃▃ | tumor microenvironment | 2015 | 13.04 | **2021** | 2025 | ▂▂▂▂▂▂▂▂▂▂▂▂▂▂▂▂▃▃▃▃▃ |
| cells | 2006 | 13.09 | **2023** | 2025 | ▂▂▂▂▂▂▂▂▂▂▂▂▂▂▂▂▂▂▃▃▃ | cells | 2006 | 13.51 | **2023** | 2025 | ▂▂▂▂▂▂▂▂▂▂▂▂▂▂▂▂▂▂▃▃▃ |

Figure S1. Keyword Co-occurrence Network and Cluster Analysis in the Dataset With Review Articles

Figure S2. Keyword Co-occurrence Network and Cluster Analysis in the Dataset Without Review Articles

Table S3. Cluster Labels and Node Distribution of Keyword Co-occurrence Networks in Datasets With and Without Review Articles

| **Research and Review** | | | **Research** | | |
| --- | --- | --- | --- | --- | --- |
| **Cluster ID** | **Label** | **Number of keywords** | **Cluster ID** | **Label** | **Number of keywords** |
| 0 | cancer immunotherapy | 51 | 0 | cancer regression | 45 |
| 1 | dendritic cells | 45 | 1 | antigen presentation | 41 |
| 2 | dna vaccine | 44 | 2 | nivolumab | 40 |
| 3 | nivolumab | 38 | 3 | responses | 40 |
| 4 | tumor microenvironment | 37 | 4 | protein | 40 |
| 5 | peptide | 33 | 5 | cross presentation | 39 |
| 6 | adoptive transfer | 32 | 6 | tumor microenvironment | 39 |
| 7 | cancer vaccine | 32 | 7 | immunization | 36 |
| 8 | lung cancer | 31 | 8 | cancer immunotherapy | 36 |
| 9 | expression | 29 | 9 | dendritic cells | 35 |
| 10 | gene therapy | 28 | 10 | ovarian cancer | 35 |
| 11 | infection | 28 | 11 | dna vaccine | 32 |
| 12 | vaccines | 27 | 12 | carcinoma | 29 |
| 13 | responses | 26 | 13 | expression | 29 |
| 14 | immunogenic cell death | 24 | 14 | cytotoxic t lymphocytes | 27 |
| 15 | breast cancer | 23 | 15 | combination therapy | 24 |
| 16 | therapy | 23 | 16 | melan-a | 21 |
| 17 | activation | 23 | 17 | gene therapy | 21 |
| 18 | melanoma | 21 |  | | |
| 19 | protein | 18 |  | | |
| 20 | t cells | 10 |  | | |

**Influence of review article inclusion on co-citation analysis**

Co-citation analysis showed that review article inclusion mainly affected the strength and connectivity of citation relationships but had limited influence on the identification of key scientific contributions.

As shown in Table S4, the top-ranked co-cited references were highly consistent between datasets. Landmark studies involving immune checkpoint blockade (Topalian et al., Hodi et al., Brahmer et al.), adoptive cell therapy (Dudley et al., Morgan et al.), tumor antigen recognition (Schumacher et al.), and personalized neoantigen vaccines (Ott et al., Sahin et al.) were repeatedly identified as core references.

Similarly, citation burst analysis demonstrated substantial overlap between datasets (Table S5). Both datasets captured the historical evolution of cancer immunotherapy, including early adoptive immunotherapy, immune checkpoint blockade, and recent neoantigen vaccine research. Although review articles contributed additional highly connected references, they did not introduce fundamentally different knowledge foundations.

**Influence of review article inclusion on co-citation clustering**

Co-citation clustering analysis showed that both datasets contained similar major research themes, including adoptive immunotherapy, antigen delivery, immune checkpoint inhibitors, T-cell receptor engineering, neoantigens, and cancer vaccines (Table S6 and Figures S3–S4).

The differences observed between clustering results mainly reflected variations in citation relationships caused by the inclusion of review articles. Since reviews often integrate knowledge from multiple research areas, their inclusion may strengthen connections among clusters. However, the main developmental trajectory—from adoptive immune cell therapy and immune checkpoint blockade toward antigen-specific and personalized immunotherapy—remained consistent.

**Overall interpretation**

Taken together, these sensitivity analyses demonstrate that review article inclusion influences citation intensity and network connectivity to some extent, but does not substantially alter the major research themes, key knowledge foundations, or evolutionary trajectory of cancer immunotherapy. Therefore, the exclusion of review articles in the primary analysis was unlikely to affect the validity of the main conclusions.

Table S4. Top 25 Most Frequently Co-cited References in Datasets With and Without Review Articles

| **Research and Review** | | | **Research** | | |
| --- | --- | --- | --- | --- | --- |
| **Rank** | **References** | **count** | **Rank** | **References** | **count** |
| 1 | Topalian SL, 2012, NEW ENGL J MED, V366, P2443, DOI 10.1056/NEJMoa1200690 | 84 | 1 | Topalian SL, 2012, NEW ENGL J MED, V366, P2443, DOI 10.1056/NEJMoa1200690 | 61 |
| 2 | Hodi FS, 2010, NEW ENGL J MED, V363, P711, DOI 10.1056/NEJMoa1003466 | 84 | 2 | Hodi FS, 2010, NEW ENGL J MED, V363, P711, DOI 10.1056/NEJMoa1003466 | 56 |
| 3 | Ott PA, 2017, NATURE, V547, P217, DOI 10.1038/nature22991 | 72 | 3 | Ott PA, 2017, NATURE, V547, P217, DOI 10.1038/nature22991 | 50 |
| 4 | Kantoff PW, 2010, NEW ENGL J MED, V363, P411, DOI 10.1056/NEJMoa1001294 | 66 | 4 | Kantoff PW, 2010, NEW ENGL J MED, V363, P411, DOI 10.1056/NEJMoa1001294 | 49 |
| 5 | Brahmer JR, 2012, NEW ENGL J MED, V366, P2455, DOI 10.1056/NEJMoa1200694 | 58 | 5 | Ribas A, 2018, SCIENCE, V359, P1350, DOI 10.1126/science.aar4060 | 46 |
| 6 | Ribas A, 2018, SCIENCE, V359, P1350, DOI 10.1126/science.aar4060 | 53 | 6 | Saxena M, 2021, NAT REV CANCER, V21, P360, DOI 10.1038/s41568-021-00346-0 | 43 |
| 7 | Morgan RA, 2006, SCIENCE, V314, P126, DOI 10.1126/science.1129003 | 53 | 7 | Schumacher TN, 2015, SCIENCE, V348, P69, DOI 10.1126/science.aaa4971 | 39 |
| 8 | Schumacher TN, 2015, SCIENCE, V348, P69, DOI 10.1126/science.aaa4971 | 53 | 8 | Palucka K, 2012, NAT REV CANCER, V12, P265, DOI 10.1038/nrc3258 | 38 |
| 9 | Saxena M, 2021, NAT REV CANCER, V21, P360, DOI 10.1038/s41568-021-00346-0 | 50 | 9 | Rosenberg SA, 2008, NAT REV CANCER, V8, P299, DOI 10.1038/nrc2355 | 37 |
| 10 | Pardoll DM, 2012, NAT REV CANCER, V12, P252, DOI 10.1038/nrc3239 | 50 | 10 | Brahmer JR, 2012, NEW ENGL J MED, V366, P2455, DOI 10.1056/NEJMoa1200694 | 37 |
| 11 | Sahin U, 2017, NATURE, V547, P222, DOI 10.1038/nature23003 | 50 | 11 | Waldman AD, 2020, NAT REV IMMUNOL, V20, P651, DOI 10.1038/s41577-020-0306-5 | 36 |
| 12 | Dudley ME, 2005, J CLIN ONCOL, V23, P2346, DOI 10.1200/JCO.2005.00.240 | 49 | 12 | Sharma P, 2015, SCIENCE, V348, P56, DOI 10.1126/science.aaa8172 | 34 |
| 13 | Keskin DB, 2019, NATURE, V565, P234, DOI 10.1038/s41586-018-0792-9 | 47 | 13 | Dudley ME, 2005, J CLIN ONCOL, V23, P2346, DOI 10.1200/JCO.2005.00.240 | 33 |
| 14 | Palucka K, 2012, NAT REV CANCER, V12, P265, DOI 10.1038/nrc3258 | 46 | 14 | Tumeh PC, 2014, NATURE, V515, P568, DOI 10.1038/nature13954 | 32 |
| 15 | Rizvi NA, 2015, SCIENCE, V348, P124, DOI 10.1126/science.aaa1348 | 45 | 15 | Gabrilovich DI, 2009, NAT REV IMMUNOL, V9, P162, DOI 10.1038/nri2506 | 31 |
| 16 | Waldman AD, 2020, NAT REV IMMUNOL, V20, P651, DOI 10.1038/s41577-020-0306-5 | 44 | 16 | Wolchok JD, 2013, NEW ENGL J MED, V369, P122, DOI 10.1056/NEJMoa1302369 | 30 |
| 17 | Wolchok JD, 2013, NEW ENGL J MED, V369, P122, DOI 10.1056/NEJMoa1302369 | 44 | 17 | Sahin U, 2017, NATURE, V547, P222, DOI 10.1038/nature23003 | 30 |
| 18 | Curiel TJ, 2004, NAT MED, V10, P942, DOI 10.1038/nm1093 | 43 | 18 | Morgan RA, 2006, SCIENCE, V314, P126, DOI 10.1126/science.1129003 | 30 |
| 19 | Sharma P, 2015, SCIENCE, V348, P56, DOI 10.1126/science.aaa8172 | 43 | 19 | Pardoll DM, 2012, NAT REV CANCER, V12, P252, DOI 10.1038/nrc3239 | 29 |
| 20 | Rosenberg SA, 2008, NAT REV CANCER, V8, P299, DOI 10.1038/nrc2355 | 42 | 20 | Dudley ME, 2008, J CLIN ONCOL, V26, P5233, DOI 10.1200/JCO.2008.16.5449 | 28 |
| 21 | Tumeh PC, 2014, NATURE, V515, P568, DOI 10.1038/nature13954 | 42 | 21 | Wculek SK, 2020, NAT REV IMMUNOL, V20, P7, DOI 10.1038/s41577-019-0210-z | 27 |
| 22 | Dudley ME, 2008, J CLIN ONCOL, V26, P5233, DOI 10.1200/JCO.2008.16.5449 | 41 | 22 | Rizvi NA, 2015, SCIENCE, V348, P124, DOI 10.1126/science.aaa1348 | 26 |
| 23 | Rosenberg SA, 2004, NAT MED, V10, P909, DOI 10.1038/nm1100 | 40 | 23 | Gattinoni L, 2006, NAT REV IMMUNOL, V6, P383, DOI 10.1038/nri1842 | 26 |
| 24 | Wculek SK, 2020, NAT REV IMMUNOL, V20, P7, DOI 10.1038/s41577-019-0210-z | 38 | 24 | Curiel TJ, 2004, NAT MED, V10, P942, DOI 10.1038/nm1093 | 26 |
| 25 | Gattinoni L, 2006, NAT REV IMMUNOL, V6, P383, DOI 10.1038/nri1842 | 37 | 25 | Johnson LA, 2009, BLOOD, V114, P535, DOI 10.1182/blood-2009-03-211714 | 26 |

Table S5. Top 25 References with the Strongest Citation Bursts in Datasets With and Without Review Articles

| **References（Research and Review）** | **2005 - 2025** | **References（Research）** | **2005 - 2025** |
| --- | --- | --- | --- |
| Rosenberg SA, 2004, NAT MED, V10, P909, DOI 10.1038/nm1100, [DOI](http://dx.doi.org/10.1038/nm1100) | ▃▃▃▃▃▂▂▂▂▂▂▂▂▂▂▂▂▂▂▂▂ | Dudley ME, 2005, J CLIN ONCOL, V23, P2346, DOI 10.1200/JCO.2005.00.240, [DOI](http://dx.doi.org/10.1200/JCO.2005.00.240) | ▂▃▃▃▃▃▂▂▂▂▂▂▂▂▂▂▂▂▂▂▂ |
| Dudley ME, 2005, J CLIN ONCOL, V23, P2346, DOI 10.1200/JCO.2005.00.240, [DOI](http://dx.doi.org/10.1200/JCO.2005.00.240) | ▂▃▃▃▃▃▂▂▂▂▂▂▂▂▂▂▂▂▂▂▂ | Curiel TJ, 2004, NAT MED, V10, P942, DOI 10.1038/nm1093, [DOI](http://dx.doi.org/10.1038/nm1093) | ▂▃▃▃▃▂▂▂▂▂▂▂▂▂▂▂▂▂▂▂▂ |
| Curiel TJ, 2004, NAT MED, V10, P942, DOI 10.1038/nm1093, [DOI](http://dx.doi.org/10.1038/nm1093) | ▂▃▃▃▃▂▂▂▂▂▂▂▂▂▂▂▂▂▂▂▂ | Morgan RA, 2006, SCIENCE, V314, P126, DOI 10.1126/science.1129003, [DOI](http://dx.doi.org/10.1126/science.1129003) | ▂▂▂▃▃▃▃▂▂▂▂▂▂▂▂▂▂▂▂▂▂ |
| Gattinoni L, 2006, NAT REV IMMUNOL, V6, P383, DOI 10.1038/nri1842, [DOI](http://dx.doi.org/10.1038/nri1842) | ▂▃▃▃▃▃▃▂▂▂▂▂▂▂▂▂▂▂▂▂▂ | Rosenberg SA, 2008, NAT REV CANCER, V8, P299, DOI 10.1038/nrc2355, [DOI](http://dx.doi.org/10.1038/nrc2355) | ▂▂▂▂▃▃▃▃▃▂▂▂▂▂▂▂▂▂▂▂▂ |
| Morgan RA, 2006, SCIENCE, V314, P126, DOI 10.1126/science.1129003, [DOI](http://dx.doi.org/10.1126/science.1129003) | ▂▂▃▃▃▃▃▂▂▂▂▂▂▂▂▂▂▂▂▂▂ | Gattinoni L, 2006, NAT REV IMMUNOL, V6, P383, DOI 10.1038/nri1842, [DOI](http://dx.doi.org/10.1038/nri1842) | ▂▂▂▂▃▃▃▂▂▂▂▂▂▂▂▂▂▂▂▂▂ |
| Rosenberg SA, 2008, NAT REV CANCER, V8, P299, DOI 10.1038/nrc2355, [DOI](http://dx.doi.org/10.1038/nrc2355) | ▂▂▂▂▃▃▃▃▃▂▂▂▂▂▂▂▂▂▂▂▂ | Dudley ME, 2008, J CLIN ONCOL, V26, P5233, DOI 10.1200/JCO.2008.16.5449, [DOI](http://dx.doi.org/10.1200/JCO.2008.16.5449) | ▂▂▂▂▂▃▃▃▃▂▂▂▂▂▂▂▂▂▂▂▂ |
| Dudley ME, 2008, J CLIN ONCOL, V26, P5233, DOI 10.1200/JCO.2008.16.5449, [DOI](http://dx.doi.org/10.1200/JCO.2008.16.5449) | ▂▂▂▂▃▃▃▃▃▂▂▂▂▂▂▂▂▂▂▂▂ | Gabrilovich DI, 2009, NAT REV IMMUNOL, V9, P162, DOI 10.1038/nri2506, [DOI](http://dx.doi.org/10.1038/nri2506) | ▂▂▂▂▂▃▃▃▃▃▂▂▂▂▂▂▂▂▂▂▂ |
| Johnson LA, 2009, BLOOD, V114, P535, DOI 10.1182/blood-2009-03-211714, [DOI](http://dx.doi.org/10.1182/blood-2009-03-211714) | ▂▂▂▂▂▃▃▃▃▃▂▂▂▂▂▂▂▂▂▂▂ | Hodi FS, 2010, NEW ENGL J MED, V363, P711, DOI 10.1056/NEJMoa1003466, [DOI](http://dx.doi.org/10.1056/NEJMoa1003466) | ▂▂▂▂▂▂▃▃▃▃▃▂▂▂▂▂▂▂▂▂▂ |
| Hodi FS, 2010, NEW ENGL J MED, V363, P711, DOI 10.1056/NEJMoa1003466, [DOI](http://dx.doi.org/10.1056/NEJMoa1003466) | ▂▂▂▂▂▂▃▃▃▃▃▂▂▂▂▂▂▂▂▂▂ | Kantoff PW, 2010, NEW ENGL J MED, V363, P411, DOI 10.1056/NEJMoa1001294, [DOI](http://dx.doi.org/10.1056/NEJMoa1001294) | ▂▂▂▂▂▂▃▃▃▃▃▂▂▂▂▂▂▂▂▂▂ |
| Kantoff PW, 2010, NEW ENGL J MED, V363, P411, DOI 10.1056/NEJMoa1001294, [DOI](http://dx.doi.org/10.1056/NEJMoa1001294) | ▂▂▂▂▂▂▃▃▃▃▃▂▂▂▂▂▂▂▂▂▂ | Topalian SL, 2012, NEW ENGL J MED, V366, P2443, DOI 10.1056/NEJMoa1200690, [DOI](http://dx.doi.org/10.1056/NEJMoa1200690) | ▂▂▂▂▂▂▂▂▃▃▃▃▃▂▂▂▂▂▂▂▂ |
| Topalian SL, 2012, NEW ENGL J MED, V366, P2443, DOI 10.1056/NEJMoa1200690, [DOI](http://dx.doi.org/10.1056/NEJMoa1200690) | ▂▂▂▂▂▂▂▂▃▃▃▃▃▂▂▂▂▂▂▂▂ | Brahmer JR, 2012, NEW ENGL J MED, V366, P2455, DOI 10.1056/NEJMoa1200694, [DOI](http://dx.doi.org/10.1056/NEJMoa1200694) | ▂▂▂▂▂▂▂▂▃▃▃▃▂▂▂▂▂▂▂▂▂ |
| Brahmer JR, 2012, NEW ENGL J MED, V366, P2455, DOI 10.1056/NEJMoa1200694, [DOI](http://dx.doi.org/10.1056/NEJMoa1200694) | ▂▂▂▂▂▂▂▂▃▃▃▃▃▂▂▂▂▂▂▂▂ | Palucka K, 2012, NAT REV CANCER, V12, P265, DOI 10.1038/nrc3258, [DOI](http://dx.doi.org/10.1038/nrc3258) | ▂▂▂▂▂▂▂▂▃▃▃▃▃▂▂▂▂▂▂▂▂ |
| Pardoll DM, 2012, NAT REV CANCER, V12, P252, DOI 10.1038/nrc3239, [DOI](http://dx.doi.org/10.1038/nrc3239) | ▂▂▂▂▂▂▂▂▃▃▃▃▃▂▂▂▂▂▂▂▂ | Rosenberg SA, 2011, CLIN CANCER RES, V17, P4550, DOI 10.1158/1078-0432.CCR-11-0116, [DOI](http://dx.doi.org/10.1158/1078-0432.CCR-11-0116) | ▂▂▂▂▂▂▂▂▃▃▃▃▂▂▂▂▂▂▂▂▂ |
| Palucka K, 2012, NAT REV CANCER, V12, P265, DOI 10.1038/nrc3258, [DOI](http://dx.doi.org/10.1038/nrc3258) | ▂▂▂▂▂▂▂▂▃▃▃▃▃▂▂▂▂▂▂▂▂ | Pardoll DM, 2012, NAT REV CANCER, V12, P252, DOI 10.1038/nrc3239, [DOI](http://dx.doi.org/10.1038/nrc3239) | ▂▂▂▂▂▂▂▂▂▃▃▃▃▂▂▂▂▂▂▂▂ |
| Wolchok JD, 2013, NEW ENGL J MED, V369, P122, DOI 10.1056/NEJMoa1302369, [DOI](http://dx.doi.org/10.1056/NEJMoa1302369) | ▂▂▂▂▂▂▂▂▂▃▃▃▃▃▂▂▂▂▂▂▂ | Wolchok JD, 2013, NEW ENGL J MED, V369, P122, DOI 10.1056/NEJMoa1302369, [DOI](http://dx.doi.org/10.1056/NEJMoa1302369) | ▂▂▂▂▂▂▂▂▂▃▃▃▃▃▂▂▂▂▂▂▂ |
| Schumacher TN, 2015, SCIENCE, V348, P69, DOI 10.1126/science.aaa4971, [DOI](http://dx.doi.org/10.1126/science.aaa4971) | ▂▂▂▂▂▂▂▂▂▂▂▃▃▃▃▃▂▂▂▂▂ | Schumacher TN, 2015, SCIENCE, V348, P69, DOI 10.1126/science.aaa4971, [DOI](http://dx.doi.org/10.1126/science.aaa4971) | ▂▂▂▂▂▂▂▂▂▂▂▃▃▃▃▃▂▂▂▂▂ |
| Tumeh PC, 2014, NATURE, V515, P568, DOI 10.1038/nature13954, [DOI](http://dx.doi.org/10.1038/nature13954) | ▂▂▂▂▂▂▂▂▂▂▂▃▃▃▃▂▂▂▂▂▂ | Tumeh PC, 2014, NATURE, V515, P568, DOI 10.1038/nature13954, [DOI](http://dx.doi.org/10.1038/nature13954) | ▂▂▂▂▂▂▂▂▂▂▂▃▃▃▃▂▂▂▂▂▂ |
| Rizvi NA, 2015, SCIENCE, V348, P124, DOI 10.1126/science.aaa1348, [DOI](http://dx.doi.org/10.1126/science.aaa1348) | ▂▂▂▂▂▂▂▂▂▂▂▃▃▃▃▃▂▂▂▂▂ | Sharma P, 2015, SCIENCE, V348, P56, DOI 10.1126/science.aaa8172, [DOI](http://dx.doi.org/10.1126/science.aaa8172) | ▂▂▂▂▂▂▂▂▂▂▂▂▃▃▃▃▂▂▂▂▂ |
| Sharma P, 2015, SCIENCE, V348, P56, DOI 10.1126/science.aaa8172, [DOI](http://dx.doi.org/10.1126/science.aaa8172) | ▂▂▂▂▂▂▂▂▂▂▂▂▃▃▃▃▂▂▂▂▂ | Ott PA, 2017, NATURE, V547, P217, DOI 10.1038/nature22991, [DOI](http://dx.doi.org/10.1038/nature22991) | ▂▂▂▂▂▂▂▂▂▂▂▂▂▃▃▃▃▃▂▂▂ |
| Ott PA, 2017, NATURE, V547, P217, DOI 10.1038/nature22991, [DOI](http://dx.doi.org/10.1038/nature22991) | ▂▂▂▂▂▂▂▂▂▂▂▂▂▃▃▃▃▃▂▂▂ | Sahin U, 2017, NATURE, V547, P222, DOI 10.1038/nature23003, [DOI](http://dx.doi.org/10.1038/nature23003) | ▂▂▂▂▂▂▂▂▂▂▂▂▂▃▃▃▃▃▂▂▂ |
| Sahin U, 2017, NATURE, V547, P222, DOI 10.1038/nature23003, [DOI](http://dx.doi.org/10.1038/nature23003) | ▂▂▂▂▂▂▂▂▂▂▂▂▂▃▃▃▃▃▂▂▂ | Ribas A, 2018, SCIENCE, V359, P1350, DOI 10.1126/science.aar4060, [DOI](http://dx.doi.org/10.1126/science.aar4060) | ▂▂▂▂▂▂▂▂▂▂▂▂▂▂▃▃▃▃▃▂▂ |
| Ribas A, 2018, SCIENCE, V359, P1350, DOI 10.1126/science.aar4060, [DOI](http://dx.doi.org/10.1126/science.aar4060) | ▂▂▂▂▂▂▂▂▂▂▂▂▂▂▃▃▃▃▃▂▂ | Spranger S, 2017, CANCER CELL, V31, P711, DOI 10.1016/j.ccell.2017.04.003, [DOI](http://dx.doi.org/10.1016/j.ccell.2017.04.003) | ▂▂▂▂▂▂▂▂▂▂▂▂▂▂▃▃▃▂▂▂▂ |
| Waldman AD, 2020, NAT REV IMMUNOL, V20, P651, DOI 10.1038/s41577-020-0306-5, [DOI](http://dx.doi.org/10.1038/s41577-020-0306-5) | ▂▂▂▂▂▂▂▂▂▂▂▂▂▂▂▂▃▃▃▃▃ | Waldman AD, 2020, NAT REV IMMUNOL, V20, P651, DOI 10.1038/s41577-020-0306-5, [DOI](http://dx.doi.org/10.1038/s41577-020-0306-5) | ▂▂▂▂▂▂▂▂▂▂▂▂▂▂▂▂▃▃▃▃▃ |
| Saxena M, 2021, NAT REV CANCER, V21, P360, DOI 10.1038/s41568-021-00346-0, [DOI](http://dx.doi.org/10.1038/s41568-021-00346-0) | ▂▂▂▂▂▂▂▂▂▂▂▂▂▂▂▂▂▃▃▃▃ | Saxena M, 2021, NAT REV CANCER, V21, P360, DOI 10.1038/s41568-021-00346-0, [DOI](http://dx.doi.org/10.1038/s41568-021-00346-0) | ▂▂▂▂▂▂▂▂▂▂▂▂▂▂▂▂▂▃▃▃▃ |
| Sung H, 2021, CA-CANCER J CLIN, V71, P209, DOI 10.3322/caac.21660, [DOI](http://dx.doi.org/10.3322/caac.21660) | ▂▂▂▂▂▂▂▂▂▂▂▂▂▂▂▂▂▂▃▃▃ | Sung H, 2021, CA-CANCER J CLIN, V71, P209, DOI 10.3322/caac.21660, [DOI](http://dx.doi.org/10.3322/caac.21660) | ▂▂▂▂▂▂▂▂▂▂▂▂▂▂▂▂▂▂▃▃▃ |

Figure S3. Co-citation Network and Cluster Analysis in the Dataset With Review Articles

Figure S4. Co-citation Network and Cluster Analysis in the Dataset Without Review Articles

Table S6. Cluster Labels and Node Distribution of Co-citation Networks in Datasets With and Without Review Articles

| **Research and Review** | | | **Research** | | |
| --- | --- | --- | --- | --- | --- |
| **Cluster ID** | **Label** | **Number of References** | **Cluster ID** | **Label** | **Number of References** |
| 0 | dendritic cell | 39 | 0 | regulatory t cells | 48 |
| 1 | adoptive immunotherapy | 39 | 1 | microneedles | 44 |
| 2 | antigen delivery | 36 | 2 | anti-tumor immunity | 40 |
| 3 | gene therapy | 35 | 3 | cytotoxic | 37 |
| 4 | checkpoint | 35 | 4 | chimeric antigen receptor | 35 |
| 5 | tcr repertoire | 33 | 5 | cd19 antigen | 31 |
| 6 | ok432 | 32 | 6 | non-small cell lung cancer | 31 |
| 7 | toll-like receptor agonist | 31 | 7 | tricom | 29 |
| 8 | t cell receptor | 31 | 8 | sipuleucel-t | 29 |
| 9 | neoantigen | 31 | 9 | pd1 | 28 |
| 10 | pd1 | 30 | 10 | prostate cancer | 27 |
| 11 | hepatocellular carcinoma | 30 | 11 | dendritic cells | 24 |
| 12 | tumor-specific t cells | 29 | 12 | cross-presentation | 22 |
| 13 | leukemia | 29 | 13 | adoptive cell therapy | 20 |
| 14 | cyclophosphamide | 28 | 14 | 3-dioxygenase | 20 |
| 15 | mrna therapeutics | 27 | 15 | cancer vaccines | 18 |
| 16 | natural killer cells | 25 | 16 | immune checkpoint inhibitor | 18 |
| 17 | renal cell carcinoma | 23 | 17 | oncolytic virus | 17 |
| 18 | immune checkpoint inhibitor | 22 | 18 | experimental autoimmune encephalomyelitis | 17 |
| 19 | b7-h1 | 22 | 19 | pd-1 | 16 |
| 20 | immune therapy | 16 | 20 | adoptive immunotherapy | 16 |
| 21 | cord blood | 14 |  | | |
| 22 | prostate-specific antigen | 12 |  | | |
